# Rapid and Intense Declines of Forest Connectivity in the Amazon Arc of Deforestation Over Four Decades

**DOI:** 10.1111/gcb.70959

**Published:** 2026-06-06

**Authors:** Mario Arthur Favretto, Marina Hirota

**Affiliations:** ^1^ Graduate Program in Ecology Federal University of Santa Catarina Florianópolis Brazil; ^2^ Instituto Relva Rio de Janeiro Brazil; ^3^ Department of Physics Federal University of Santa Catarina Florianópolis Brazil

**Keywords:** Amazon deforestation arc, dispersal thresholds, forest fragmentation, functional connectivity, landscape resilience

## Abstract

The Amazon Arc of Deforestation is facing a silent decline in ecological functionality. Using four decades of high‐resolution land cover data, we reveal a dramatic decline in functional connectivity across deforested areas in the Brazilian Amazon. Analyzing 40 targeted large‐scale forest landscapes across Pará, Mato Grosso, and Rondônia, we quantified connectivity for key ecological processes—seed dispersal, pollen dispersal, and gap‐crossing capacity of animals—using graph theory metrics. Between 1990 and 2020, the connectivity dropped by up to 50% in highly deforested regions, even when accounting for stepping‐stones. Simultaneously, spatial modularity increased in nearly all study regions, in some by more than 25%, reflecting a more compartmentalized landscape structure. Forest fragment distances rose, and mean patch area fell sharply, compromising dispersal and potentially gene flow. Our results suggest a potential breakdown of spatial connectivity within the targeted zones of the Amazonian forests, except in the Western Pará region, where higher connectivity and lower modularity still prevail. Without urgent connectivity‐enhancing strategies, even remaining forest patches may become functionally isolated, threatening the maintenance of biodiversity.

## Graphical abstract

Deforestation increased landscape fragmentation between 1990 and 2020. Ecological distance thresholds constrain gap crossing, seed dispersal, and pollen dispersal. Functional connectivity declined over time, while landscape modularity increased.

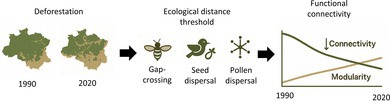

## 1 Introduction

The Brazilian Amazon remained largely preserved until the 1970s, when large‐scale development policies, such as the construction of the Trans‐Amazonian Highway, triggered widespread deforestation across the region (Fearnside 2005, 2021). In the following decades, forest loss accelerated until the early 2000s, when stricter environmental policies contributed to a temporary reduction in deforestation rates (Fearnside 2005; Matricardi et al. 2020; Fearnside 2021). However, in recent years, new cycles of land conversion and deforestation have emerged, reflecting deeper socio‐economic and political drivers of environmental exploitation (Gatti et al. 2023).

These transformations have resulted not only in extensive forest loss but also in severe fragmentation of the remaining habitats (Fearnside 2005; Fearnside 2021). Forest fragmentation disrupts ecological connectivity by isolating patches and interrupting continuous forest cover, often initiated by road construction, which typically precedes and facilitates deforestation in the Brazilian Amazon (Collinge 2009). Although such fragmentation reduces structural connectivity, which consists of the physical continuity between habitat patches, functional connectivity may nonetheless persist despite habitat fragmentation (With 2019).

Functional connectivity refers to the ability of organisms—such as animals, seeds, or pollen—to disperse across the landscape and maintain ecological processes despite structural disconnection (Collinge 2009; With 2019). For example, some species can cross deforested areas, facilitating gene flow and dispersal events (Claramunt et al. 2022). Even when habitat patches are physically separated, functional connectivity can preserve essential ecosystem functions such as reproduction, colonization, and population persistence (Fahrig et al. 2022).

The Amazon forests play a critical role in regulating climate through carbon storage and water recycling, while also harboring unparalleled levels of biodiversity (Albert et al. 2023; Yao et al. 2024; Cui et al. 2026). Beyond regional impacts, the biome exerts global influence by stabilizing atmospheric circulation and modulating precipitation patterns across South America and even other continents (Nobre et al. 2016; Franco et al. 2025). Consequently, deforestation and habitat degradation threaten not only local ecological processes but also the resilience of the Earth system as a whole.

From a conservation standpoint, maintaining functional connectivity has become a central challenge. The collapse of ecological linkages among forest patches threatens mutualistic interactions such as pollination and seed dispersal, undermining forest regeneration and long‐term ecosystem stability (Emer et al. 2018). Despite the recognition of connectivity as a key element for biodiversity conservation, current environmental governance in the Brazilian Amazon still struggles to incorporate it into protected area design and infrastructure planning (Castro et al. 2020). Understanding how connectivity has changed over time is, therefore, crucial for informing management strategies and preventing the biome from approaching critical tipping points.

In the Amazon, studies evaluating connectivity under different land‐use conditions have been widely applied to highlight zones that should be prioritized for conservation or restoration actions (Santos et al. 2019; Castro et al. 2020; Miranda et al. 2021). A central element in these assessments is the dispersal distance threshold, which determines the spatial scale at which patches are considered ecologically connected (Taylor et al. 1993; Urban and Keitt 2001). However, these distance thresholds are frequently defined arbitrarily, often relying on dispersal information from a small subset of species, and in some cases, species originating from entirely different biomes or continents (Santos et al. 2019; Castro et al. 2020; Miranda et al. 2021). This methodological approach leads to connectivity estimates that do not accurately reflect the dispersal ecological characteristics of Amazonian taxa.

More recent analyses have continued to adopt arbitrary dispersal distances while additionally relying on coarse‐resolution land‐cover datasets, which, despite enabling broad‐scale evaluations, obscure critical details about forest fragmentation patterns (Ritter et al. 2025; Teixeira et al. 2025). Consequently, to date, there are no studies of forest connectivity in the Brazilian Amazon that incorporate empirically grounded dispersal information from regional species together with high‐resolution land‐cover data.

Here, we examine long‐term changes in functional connectivity and modularity in deforested areas of the Amazon forests, particularly regions that have been experiencing some of the highest rates of forest loss on the planet (Mapbiomas 2022). We aim to evaluate the erosion of connectivity in this critical biome between the 1990s and the 2020s. Using patch and landscape metrics and ecological thresholds associated with pollen, seed, and animal dispersal, we assessed how landscape structure and connectivity have changed over the past four decades.

## 2 Materials and Methods

### 2.1 Study Areas

**FIGURE 1:**
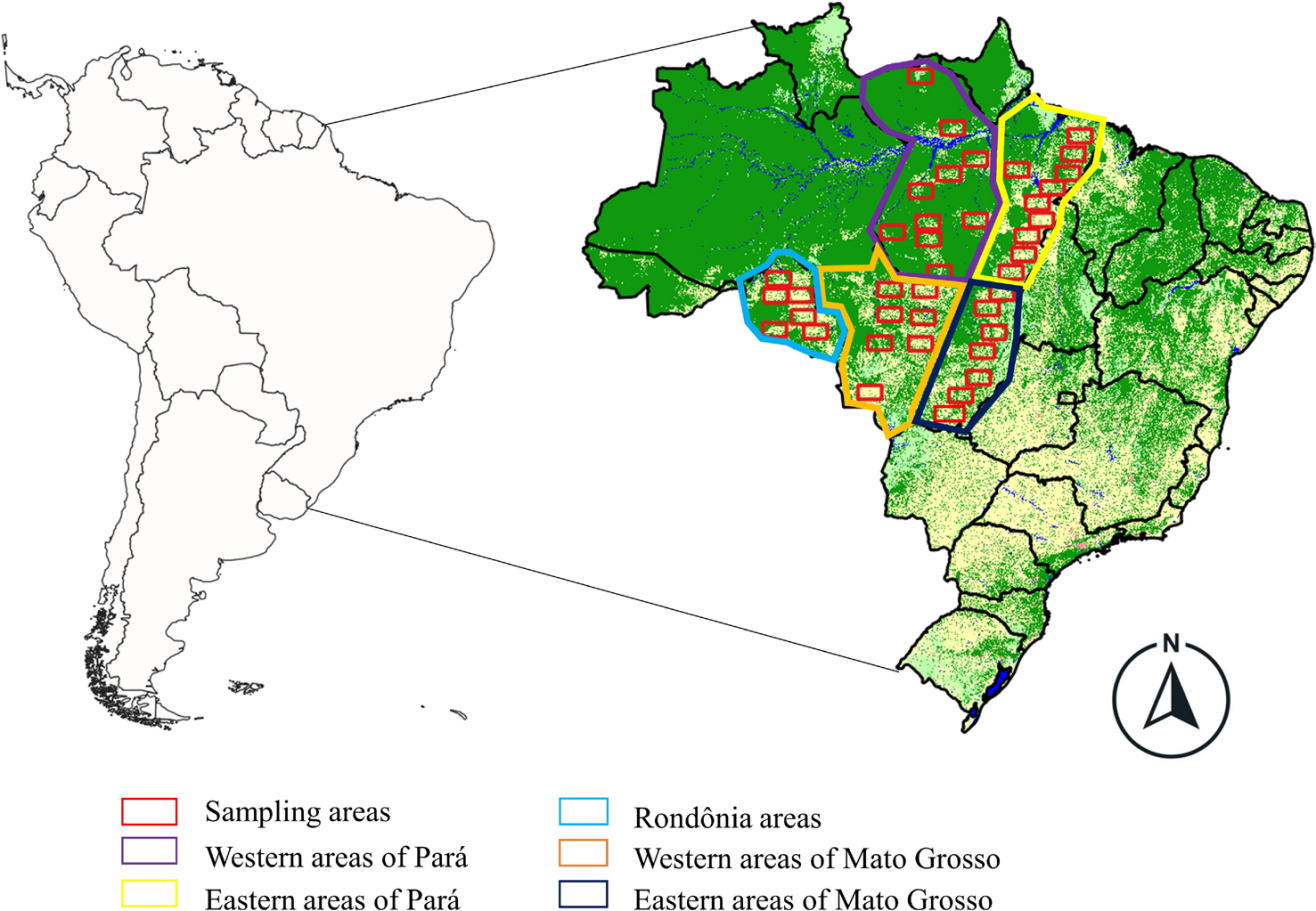
Geographic location of the study areas (red rectangles) across the states of Pará, Mato Grosso, and Rondônia in the Brazilian Amazon, South America. Study regions were selected based on varying degrees (regions) of forest fragmentation to assess functional connectivity under different levels of anthropogenic disturbance. Map lines delineate study areas and do not necessarily depict accepted national boundaries.

The overall study region comprises deforested areas of some of Amazon Arc of Deforestation states, that is, Pará, Mato Grosso, and Rondônia, states that present among the highest rates of deforestation (Mapbiomas 2022). Along these states, we visually selected study areas under forest fragmentation and deforestation, the focus of our assumptions and objectives to investigate how connectivity patterns are changing over time, given the biological information on Amazonian species. We used data from 1990, 2000, 2010, and 2020 to characterize a decadal time interval to evaluate changes in connectivity and modularity (Collection 6; Mapbiomas 2022). Moreover, we visually defined areas into possibly *more* and *less* deforested regions (Figure 1) to account for different levels of forest fragmentation.

The number of study areas (red rectangles in Figure 1) is set as follows: Western Pará state (less deforested, *n* = 10); Eastern Pará state (more deforested, *n* = 10); Western Mato Grosso state (less deforested, *n* = 7); Eastern Mato Grosso state (more deforested, *n* = 7); and Rondônia state (*n* = 6) (Mapbiomas 2022). Rather than relying on random or systematic sampling, we adopted a targeted stratified design (Gardener 2017) to ensure representation of ecologically meaningful contrasts in fragmentation intensity across the Amazon Arc of Deforestation. Given the size of Rondônia state compared to Pará and Mato Grosso, we did not separate between more and less deforested areas, but only covered all the areas that have undergone deforestation to some extent. The extraction of information from these land use sections was performed using QGis software, version 3.16.16.

The number of areas sampled varied by state due to the difference in extent of each state, with each sampling area (red rectangle) having 1,148,000 ha, the maximum size that our computational capacity was able to evaluate, considering the high spatial resolution (30 × 30 m pixels).

### 2.2 Patch and Landscape Metrics

To verify how forest fragmentation is affecting functional connectivity in these regions, spatial structure metrics were calculated at 30 m spatial resolution (Mapbiomas 2022) as input for the software FragStat, version 4.2 (McGarigal et al. 2012). The Euclidean nearest neighbor distance was used to verify the current distance between forest fragments. In addition, we computed the fragment area and the shape index, which, together with the distance, can influence structural connectivity (With 2019). The shape index consists of a ratio between the perimeter and the area of the fragment: the higher the value, the more irregular the shape of a fragment, and the lower the more regular (circular/quadrangular) the shape (McGarigal et al. 2012).

For functional connectivity, we adopted dispersal and movement distance thresholds based on the synthesis proposed by Favretto and Hirota (2026), which compiles empirical estimates relevant to functional connectivity in Amazonian forests. Following this framework, we applied biologically informed thresholds that reflect realistic dispersal capabilities for pollen (average maximum distance of 1530 m), seed dispersal (average maximum distance of 490 m), and the capacity of animals to cross open areas between forest fragments (hereafter referred to as gap‐crossing capacity; average maximum distance of 310 m), grounded in regional species traits and ecological behavior. This approach enhances the ecological relevance of the connectivity metrics used, allowing for more accurate inference about landscape functioning.

These functional connectivity distance thresholds were projected onto a graph, generating links between different nodes that represented the different forest fragments, based on Euclidean connection processes. In this way, the connectivity between the different environments under analysis (i.e., forest remnants) was determined by the proximity of other fragments of the same vegetation type that are at distances smaller than the defined thresholds.

Data were also analyzed using the probability of connectivity (PC) metric, which quantifies the degree of connectivity between habitat patches by incorporating not only direct linear links but also the stepping‐stone effect, whereby patches can be connected through intermediate areas that facilitate species movement (He et al. 2018; With 2019). As a result, the path that maximizes connectivity is not necessarily the shortest one (Saura and Fuente 2017). This metric enables comparisons of functional connectivity levels among different landscape sections, indicating relatively higher or lower connectivity (Duane et al. 2021). In practical terms, PC can be defined as “the probability that two animals randomly placed within the landscape fall into habitat areas that are reachable from each other” (Rivas and Navarro‐Cerrillo 2024). The equation defining PC is:

$$\mathrm{PC}=\frac{{\Sigma_{i=1}^{n}\Sigma }_{j=1}^{n}a_{i}{a_{j}p}_{\mathrm{ij}}^{*}}{A_{L}^{2}},$$

where the coefficients *a*_*i*_ and *a*_*j*_ are the areas of the patches, A_L_ is the total area of the landscape, and p*_ij_ is the maximum value of the product of the probabilities of each of the connections between patches *i* and *j*, considering all possible dispersal paths linking them. Therefore, each path is defined as a sequence of steps between patches, and the probability of a given path is calculated as the product of the dispersal probabilities associated with each step along that path. The value of p*_ij_ therefore, corresponds to the highest product probability among all direct and indirect paths between *i* and *j*. When patches are sufficiently close, the maximum‐probability path is the direct connection and p*_ij_ = *p*_*ij*_. For more distant patches, indirect paths through intermediate stepping‐stone patches may yield higher probabilities than the direct connection, resulting in p*_ij_>*p*_*ij*_. If no path exists between two patches, p*_ij_ = 0, while for *i = j*, p*_ij_ = 1, reflecting the habitat availability concept whereby a patch is always fully connected to itself (Saura and Pascual‐Hortal 2007).

Modularity (Q) is a widely used metric for quantifying the strength of modular structure in networks, measuring how much a given partition of a network into graphs deviates from what would be expected under a random null model. Formally, modularity corresponds to the difference between the fraction of edges connecting vertices within the same graph and the fraction of such edges expected in a random network with the same degree distribution and can be defined as

$$Q=\sum_{i}\left(e_{\mathrm{ii}}-a_{i}^{2}\right),$$

where *e*_*ii*_ represents the proportion of edges internal to graph *i* and *a*_*i*_ is the fraction of edge ends associated with that graph. High values of Q indicate a more pronounced modular organization, characterized by dense internal connectivity and sparse connections between graphs (Newman 2006; Brandes et al. 2008). Probability of connectivity and modularity were performed using Graphab software, version 2.8 (Foltête et al. 2021).

The percentage of forest cover was calculated as the proportional relationship between the total area (in hectares) of the sampling sites and the total forested area (in hectares) for each year and sampled region. This metric was obtained by dividing the forested area by the total sampled area and multiplying the result by 100, thereby expressing forest cover as a percentage for each spatial and temporal unit analyzed.

### 2.3 Statistical Analysis

The results of the structural characteristics of the forest fragments (distance, area, and shape index), probability of connectivity, and modularity were analyzed spatially and temporally using Linear Mixed Models, with the likelihood ratio test method. We used the metrics as dependent variables; year and region as fixed effects; and study areas as a random effect. These analyses were made in the software JASP, version 0.17 (JASP Team 2023).

## 3 Results

### 3.1 Structural Attributes of Forest Fragments

**FIGURE 2:**
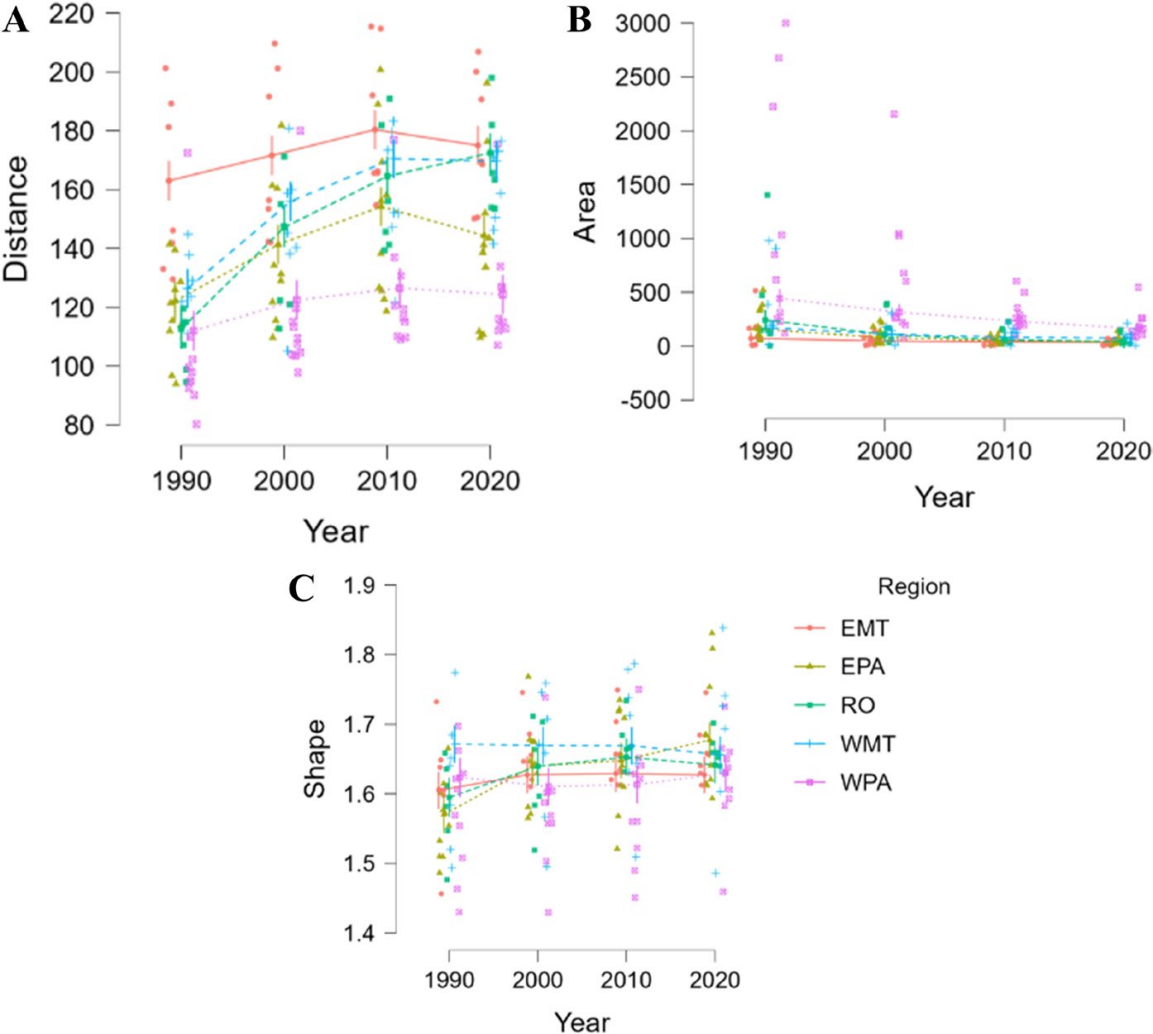
Temporal variation in the structural characteristics of forest fragments in deforested areas of the Amazon forests (1990–2020), including: Distance between forest fragments (A), mean area of forest fragments (B), and shape of forest fragments (C). The results show a progressive increase in distance and reduction in fragment area, as well as greater geometric irregularity, indicating intensified fragmentation and its effects on structural connectivity. Region: EMT, Eastern Mato Grosso state; EPA, Eastern Pará state; RO, Rondônia state; WMT, Western Mato Grosso state; WPA, Western Pará state.

The mean distance between forest fragments increased in most regions from 1990 to 2020 (LMM years: *χ* ^2^ = 5348.66; *p* < 0.001; LMM regions: *χ* ^2^ = 9576.30; *p* < 0.001; Figure 2A; Table 1). The largest relative increase occurred in Rondônia, with more than 50% increase in mean distance. Western Mato Grosso (WMT) and Eastern Pará (EPA) also showed substantial increases of ~31% and ~19%, respectively. Only Western Pará (WPA) exhibited relatively stable distances over time.

**TABLE 1:** Structural characteristics in mean values by region and study year in deforested areas of the Amazon forests. It includes values for average distance between fragments, average area, and shape index, as well as absolute and percentage differences between 1990 and 2020. The data highlight sharp reductions in area and increases in distance between fragments in Rondônia and Eastern Pará. Region: EMT, Eastern Mato Grosso state; EPA, Eastern Pará state; RO, Rondônia state; WMT, Western Mato Grosso state; WPA, Western Pará state.

Structural metric	Region	Study year				Difference 1990–2020	Percentual difference
		1990	2000	2010	2020		
Average distance between forest fragments	EMT	165.84 m	172.39 m	179.64 m	173.45 m	7.6 m	4.58%
	WMT	125.66 m	153.44 m	166.49 m	164.77 m	39.1 m	31.12%
	EPA	121.55 m	141.73 m	154.65 m	145 m	23.45 m	19.29%
	WPA	105.89 m	115.95 m	122.26 m	122.47 m	16.58 m	15.66%
	RO	112.25 m	146.75 m	163.72 m	170.46 m	58.21 m	51.86%
Average forest fragments area	EMT	64.91 ha	43.62 ha	33.57 ha	30.39 ha	−34.52 ha	−53.18%
	WMT	148.85 ha	96.28 ha	72.27 ha	62.21 ha	−86.64 ha	−58.21%
	EPA	148.59 ha	76.81 ha	45.99 ha	35.64 ha	−112.95 ha	−76.02%
	WPA	440.81 ha	313.54 ha	226.53 ha	158.36 ha	−282.45 ha	−64.08%
	RO	249.57 ha	114.61 ha	65.19 ha	47.33 ha	−202.25 ha	−81.04%
Shape index	EMT	1.62	1.65	1.65	1.65	0.03	1.69%
	WMT	1.7	1.7	1.7	1.68	−0.01	−0.65%
	EPA	1.58	1.64	1.65	1.67	0.1	6.18%
	WPA	1.62	1.62	1.62	1.64	0.01	0.85%
	RO	1.61	1.66	1.67	1.66	0.05	3.02%
Forest cover	EMT	41.68%	29.94%	23.86%	23.19%	17.26%	—
	WMT	66.38%	48.27%	37.89%	36.49%	35.25%	—
	EPA	72.95%	52.18%	39.50%	37.70%	29.89%	—
	WPA	90.43%	85.83%	78.53%	73.16%	18.50%	—
	RO	78.32%	58.07%	43.07%	37.25%	41.07%	—

All regions experienced a reduction in average forest fragment area, with the most severe declines in Rondônia (RO: −81%) and Eastern Pará (EPA: −76%) (LMM years: *χ* ^2^ = 55.98; *p* < 0.001; LMM regions: *χ* ^2^ = 113.79; *p* < 0.001; Figure 2B; Table 1). Despite its relative temporal stability in average distances among fragments, Western Pará (WPA) has also presented a reduction in mean fragment area of ~64% over the 40 years.

Forest fragment shape index, a measure of geometric complexity, increased in most regions, particularly in Eastern Pará (+6.18%) and Rondônia (+3.02%), suggesting more irregularly shaped fragments over time (LMM years: *χ* ^2^ = 122.75; *p* < 0.001; LMM regions: *χ* ^2^ = 211.36; *p* < 0.001; Figure 2C; Table 1), indicating intensified fragmentation and its effects on structural connectivity. Western Mato Grosso was the only region where the index slightly decreased.

**FIGURE 3:**
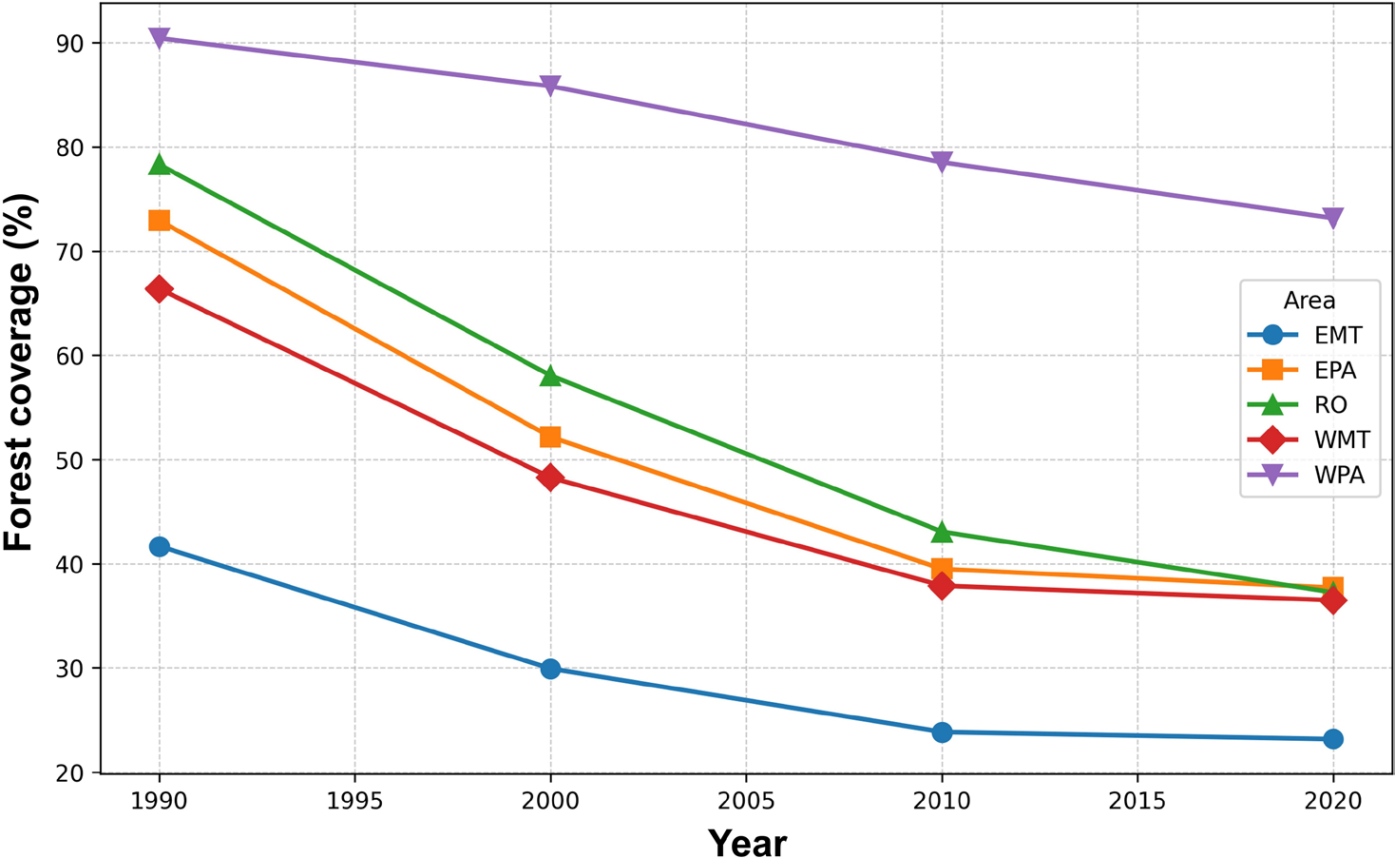
Temporal variation in the percentual coverage of forests in deforested areas of the Amazon sampling regions (1990–2020). The results show a progressive decrease in forest coverage in all sampling regions. Region: EMT, Eastern Mato Grosso state; EPA, Eastern Pará state; RO, Rondônia state; WMT, Western Mato Grosso state; WPA, Western Pará state.

Forest cover declined consistently across all regions between 1990 and 2020, although the magnitude of this reduction varied spatially (Figure 3). Eastern Mato Grosso (EMT) exhibited the lowest forest cover throughout the entire period, along with one of the most pronounced proportional declines, reflecting intense land‐use change. In contrast, western Pará (WPA) maintained the highest levels of forest cover, despite also experiencing a steady decrease over time. Intermediate regions such as eastern Pará (EPA), Rondônia (RO), and western Mato Grosso (WMT) showed moderate values but followed similar downward trends.

### 3.2 Decline of Functional Connectivity

**FIGURE 4:**
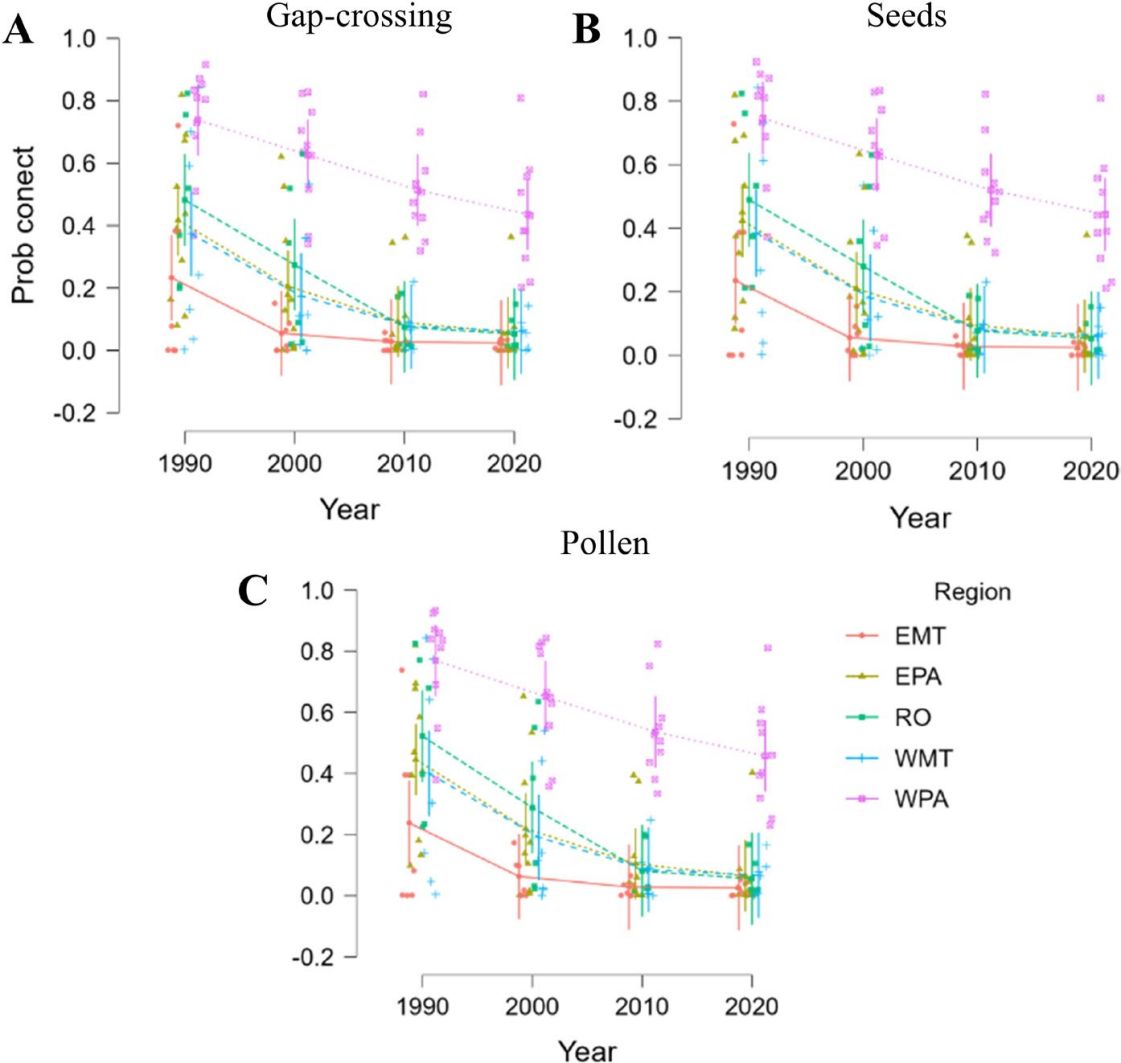
Temporal variation of the probability of connectivity (prob connect) in deforested areas of the Amazon forests, using three distance thresholds that represent functional connectivity, gap‐crossing (310 m; A), seed dispersal (490 m; B), and pollen dispersal (1530 m; C). There has been a widespread decline in connectivity, especially in Rondônia and Mato Grosso, while Western Pará maintains higher values. Region: EMT, Eastern Mato Grosso state; EPA, Eastern Pará state; RO, Rondônia state; WMT, Western Mato Grosso state; WPA, Western Pará state.

Across all three functional distance thresholds we observed a remarkable decline in the probability of connectivity (PC) from 1990 to 2010, followed by a relative stabilization between 2010 and 2020 (Figure 4; Table 2; LMM: Gap‐crossing [310 m]: *χ* ^2^ = 70.4, *p* < 0.001; seed dispersal [490 m]: *χ* ^2^ = 71.18, *p* < 0.001; pollen dispersal [1530 m]: *χ* ^2^ = 73.90, *p* < 0.001). Also, with significant differences between regions (LMM: Gap‐crossing [310 m]: *χ* ^2^ = 125.66, *p* < 0.001; seed dispersal [490 m]: *χ* ^2^ = 125.86, *p* < 0.001; pollen dispersal [1530 m]: *χ* ^2^ = 127.17, *p* < 0.001).

**TABLE 2:** Average probability of connectivity (PC) in percentage values by distance threshold, region and study year in deforested areas of the Amazon forests. It presents the percentage values of functional connectivity for gap‐crossing, seed dispersal, and pollen dispersal, highlighting the decline of more than 40% in Rondônia between 1990 and 2020. Region: EMT, Eastern Mato Grosso state; EPA, Eastern Pará state; RO, Rondônia state; WMT, Western Mato Grosso state; WPA, Western Pará state.

Distance threshold	Region	Study year				Difference 1990–2020
		1990	2000	2010	2020	
Gap‐crossing	EMT	22.34%	4.55%	1.82%	1.43%	−20.92%
	WMT	36.37%	16.47%	6.77%	5.14%	−31.23%
	EPA	41.75%	20.53%	9.25%	5.70%	−36.05%
	WPA	73.84%	62.60%	51.37%	43.64%	−30.20%
	RO	47.92%	27.14%	7.20%	4.93%	−42.99%
Seed dispersal	EMT	22.61%	4.68%	1.89%	1.55%	−21.06%
	WMT	37.57%	17.13%	7.11%	5.36%	−32.21%
	EPA	42.37%	20.97%	9.64%	5.96%	−36.41%
	WPA	74.63%	63.14%	52.05%	44.33%	−30.30%
	RO	48.69%	27.74%	7.46%	5.07%	−43.62%
Pollen dispersal	EMT	23.05%	5.56%	2.09%	1.85%	−21.21%
	WMT	39.29%	18.34%	7.79%	5.91%	−33.38%
	EPA	44.57%	21.96%	10.39%	6.51%	−38.05%
	WPA	76.96%	65.16%	53.63%	45.76%	−31.20%
	RO	52.21%	28.83%	8.12%	5.52%	−46.70%

Among the five regions analyzed, Rondônia (RO) experienced the steepest reduction in PC, with values declining from approximately 50% in 1990 to below 5% in 2020. Eastern Mato Grosso (EMT) currently shows the lowest overall PC values across all distance thresholds, decreasing from ~22% to < 2%. In contrast, Western Pará (WPA) retained the highest connectivity values throughout the period, though it has also experienced substantial losses (from ~75% to ~45%).

### 3.3 Increase in Spatial Modularity

**FIGURE 5:**
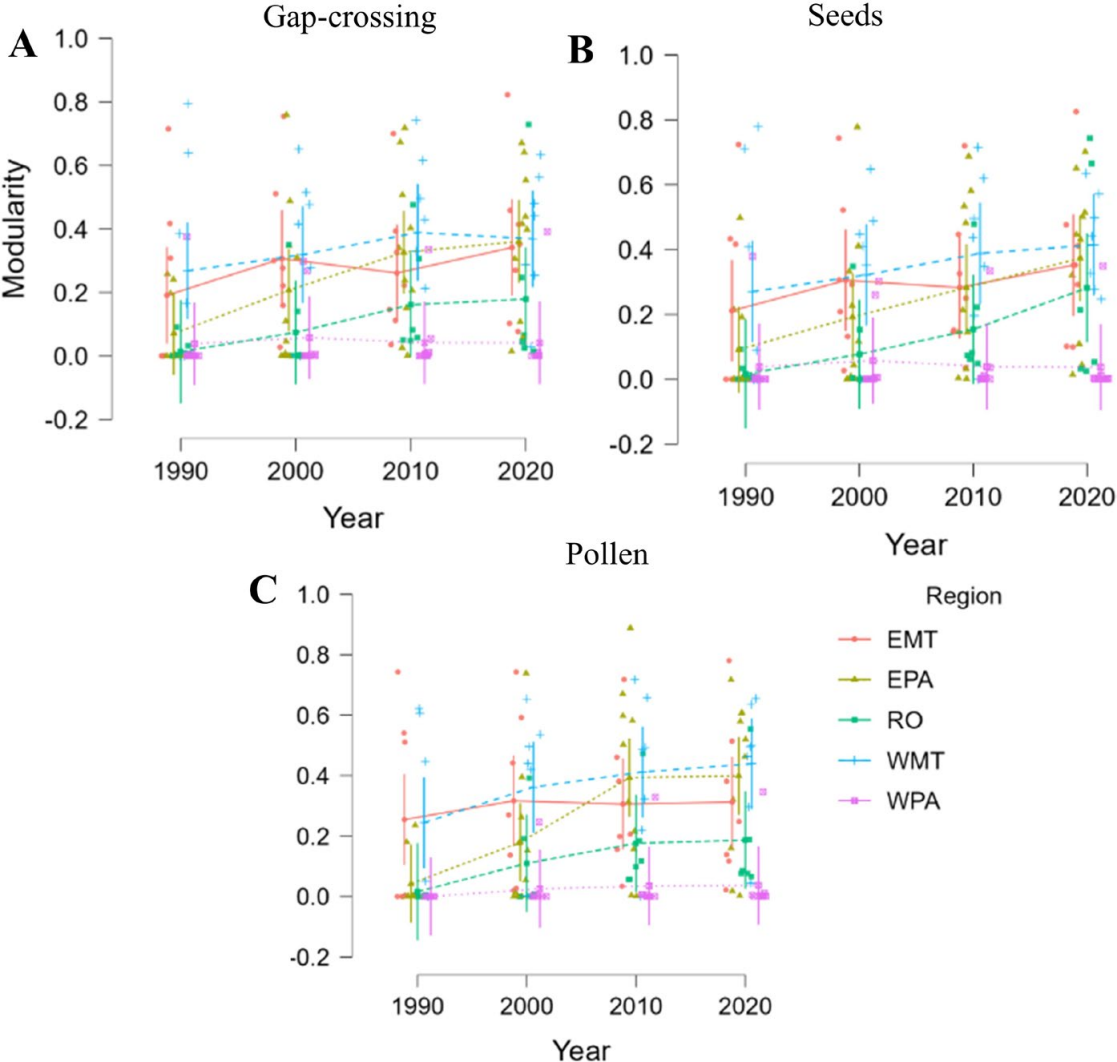
Temporal variation of spatial modularity in deforested areas of the Amazon forests, using three distance thresholds that represent functional connectivity, gap‐crossing (310 m; A), seed dispersal (490 m; B) and pollen dispersal (1530 m; C). The growth in modularity indicates larger compartmentalization of the landscape, particularly in Eastern Pará and Rondônia, suggesting that fragmentation promotes more isolated connectivity networks. Region: EMT, Eastern Mato Grosso state; EPA, Eastern Pará state; RO, Rondônia state; WMT, Western Mato Grosso state; WPA, Western Pará state.

Modularity increased over time in nearly all regions and for all distance thresholds, indicating a shift towards more compartmentalized spatial structures, suggesting that fragmentation promotes more isolated connectivity networks (Figure 5; Table 3). The most pronounced increases were observed in Eastern Pará (EPA) and Rondônia (RO), where modularity values rose by more than 25% between 1990 and 2020 (LMM: Gap‐crossing [310 m]: *χ* ^2^ = 14.11, *p* = 0.003; seed dispersal [490 m]: *χ* ^2^ = 15.96, *p* = 0.001; pollen dispersal [1530 m]: *χ* ^2^ = 21.34, *p* < 0.001). In contrast, Western Pará (WPA) maintained consistently low modularity values across all years, suggesting a more connected forest landscape. Also, with significant differences between regions (LMM: Gap‐crossing [310 m]: *χ* ^2^ = 51.62, *p* < 0.001; seed dispersal [490 m]: *χ* ^2^ = 49.11, *p* < 0.001; pollen dispersal [1530 m]: *χ* ^2^ = 67.94, *p* < 0.001).

**TABLE 3:** Average modularity in percentage values by distance threshold, region and study year in deforested areas of the Amazon forests. It shows how the structure of connectivity networks has become more compartmentalized over four decades, with increases of up to 35% in highly deforested regions. Region: EMT, Eastern Mato Grosso state; EPA, Eastern Pará state; RO, Rondônia state; WMT, Western Mato Grosso state; WPA, Western Pará state.

Distance threshold	Region	Study year				Difference 1990–2020
		1990	2000	2010	2020	
Gap‐crossing	EMT	20.59%	32.14%	27.63%	35.65%	15.05%
	WMT	28.35%	33.40%	40.37%	38.39%	10.04%
	EPA	7.01%	20.69%	32.64%	36.03%	29.02%
	WPA	3.86%	5.76%	4.14%	4.13%	0.27%
	RO	2.23%	8.25%	17.00%	18.76%	16.53%
Seed dispersal	EMT	22.50%	31.89%	29.66%	36.57%	14.07%
	WMT	28.40%	33.55%	40.17%	42.82%	14.43%
	EPA	9.11%	19.20%	28.26%	37.34%	28.23%
	WPA	3.85%	5.74%	3.89%	3.70%	−0.16%
	RO	2.32%	8.42%	16.10%	28.89%	26.57%
Pollen dispersal	EMT	25.66%	31.86%	30.81%	31.45%	5.80%
	WMT	24.68%	36.41%	41.44%	44.15%	19.47%
	EPA	4.21%	17.96%	39.33%	39.96%	35.75%
	WPA	0.00%	2.54%	3.47%	3.70%	3.70%
	RO	0.43%	9.82%	16.45%	17.47%	17.04%

## 4 Discussion

Our results indicate that fragmentation in the Amazon is not merely reducing connectivity but actively reorganizing the landscape into semi‐isolated functional modules, within which ecological processes may persist locally but are increasingly decoupled at the landscape scale. Also, our findings reveal a striking and continuous decline in functional connectivity across deforested areas of the Amazon forests over the past four decades. Despite the presence of stepping‐stones (small forest patches that may facilitate movement across fragmented landscapes), functional connectivity declined sharply in several regions, with average probabilities of connectivity dropping below 10% in all study regions, except Western Pará. This pattern indicates that, even where structural elements that could theoretically support movement persist, ecological flows are increasingly constrained. Such a reduction points to a systemic breakdown in the spatial functionality of these landscapes, consistent with fragmentation‐driven disruptions documented for Amazon forests (Laurance et al. 2000; Hooper and Ashton 2020).

The consistent decline in forest cover across all regions between 1990 and 2020 reflects the widespread and persistent nature of land‐use change in the Amazonian Arc of deforestation (Laurance et al. 2001). The markedly lower values of forest coverage observed in eastern Mato Grosso (EMT) reinforce its characterization as one of the most heavily transformed regions, historically driven by agricultural expansion, particularly cattle ranching and soybean cultivation (Fearnside 2005). In contrast, western Pará (WPA) maintained comparatively high levels of forest cover, suggesting a later or less intense occupation process, although the observed decline indicates that these areas are increasingly subject to similar pressures. The intermediate trajectories observed in eastern Pará (EPA), Rondônia (RO), and western Mato Grosso (WMT) highlight the spatial heterogeneity of deforestation dynamics, likely influenced by differences in infrastructure development, accessibility, and policy enforcement (Fearnside 2005; Barona et al. 2010; Arima et al. 2011). Importantly, the overall downward trend across all regions suggests that forest loss is not restricted to boundary zones but is expanding into previously less disturbed areas.

The observed decline in functional connectivity has direct and far‐reaching implications for dispersal‐related ecological processes. Functional connectivity underpins gene flow, seed dispersal, pollination, and population viability by enabling organisms to move among habitat patches (Fahrig et al. 2022). As inter‐fragment distances increase and patch areas shrink, these processes become progressively restricted, leading to spatially isolated populations, reduced genetic diversity, and increased local extinction risk (Smith and Pauli 2024). In highly fragmented landscapes, even dispersal mechanisms that are relatively tolerant to habitat discontinuity may become ineffective, particularly when fragment sizes fall below thresholds required to sustain viable populations (Fahrig 2003; Hanski 2011). These risks are further intensified by the combined effects of increased isolation and reduced habitat area (Ferraz et al. 2003).

Small and isolated forest fragments are also disproportionately affected by edge effects, which alter microclimatic conditions and biotic interactions (Murcia 1995). Increased desiccation, temperature variability, and wind exposure can reduce habitat suitability, while biotic degradation, such as the loss of interior forest species and the proliferation of generalist or disturbance‐adapted taxa, further compromises ecological functioning (Collinge 2009; Siegel et al. 2024). Together, these processes limit the capacity of fragments to act as effective stepping‐stones, thereby accelerating the erosion of functional connectivity.

Regional contrasts highlight that the erosion of functional connectivity is not uniform across the Amazon Arc of Deforestation. Rondônia and Eastern Pará exhibited the steepest declines, reflecting their longer histories of agricultural expansion, denser road networks, and higher population pressure (Yoshikawa and Sanga‐Ngoie 2011; Du et al. 2025). In contrast, Western Pará consistently maintained higher functional connectivity and lower modularity, likely due to later frontier expansion and lower cumulative deforestation intensity (Yoshikawa and Sanga‐Ngoie 2011; Du et al. 2025). These contrasting trajectories suggest that landscape configuration, historical land‐use dynamics, and regional socio‐economic drivers jointly shape the degree to which dispersal processes can persist in fragmented environments (Turner and Gardner 2015).

The temporal scale of these changes is particularly concerning. The collapse of functional connectivity occurred within less than four decades, a period that is extremely short in ecological terms. Such rapid erosion of dispersal processes suggests that Amazon forests may be approaching critical functional thresholds faster than other tropical biomes, such as the Atlantic Forest, where fragmentation has occurred over longer timescales (Galán‐Acedo et al. 2023; Passel et al. 2024). This rapid pace reduces opportunities for ecological adjustment and emphasizes the urgency of proactive, rather than reactive, landscape management.

Landscapes may retain substantial forest cover while already being functionally fragmented, with dispersal‐related processes such as gene flow, pollination, and seed dispersal operating at critically reduced levels (Hooper and Ashton 2020). This decoupling between structural and functional connectivity underscores a key limitation of conservation strategies based solely on forest cover metrics, highlighting the hidden vulnerability of landscapes that appear structurally intact but are functionally degraded (Imong et al. 2014; Issii et al. 2020).

The loss of functional connectivity also has cascading effects on mutualistic networks. Plant‐pollinator and plant‐seed disperser interactions depend on the movement of organisms across the landscape, and their disruption can directly impair forest regeneration and long‐term ecosystem stability (Ferreira et al. 2020; Siegel et al. 2024). As connectivity declines, specialist species are often lost first, leading to a shift toward simplified interaction networks dominated by generalist species (Tylianakis et al. 2008; Didham et al. 2012). These generalized interactions are typically less efficient and less resilient, further undermining ecosystem functioning (Vitali et al. 2023).

The observed increase in spatial modularity provides additional insight into the nature of this fragmentation process. While moderate modularity can enhance resilience by containing disturbances within clusters, the concurrent increase in modularity and decline in inter‐module connectivity observed here suggests growing spatial compartmentalization (Cumming 2011; Scheffer et al. 2012; Liu et al. 2022). In regions such as Eastern Pará, high modularity combined with low overall connectivity implies that forest remnants are increasingly organized into semi‐isolated modules, within which ecological processes may persist locally but with limited exchange among modules (Urban and Keitt 2001). This configuration reduces landscape‐scale robustness and constrains recovery following local disturbances (With 2019).

Our biologically informed distance thresholds allow a more realistic interpretation of which organisms are likely to sustain dispersal processes in fragmented Amazon landscapes. Gap‐crossing distances of approximately 150–310 m are beyond the routine movement capacity of most forest‐dependent birds, small mammals, and understory species, which typically avoid open matrices (Offerman et al. 1995; Julliot 1996; Lees and Peres 2009). Under these conditions, functional connectivity is likely maintained by a restricted subset of highly mobile generalists, such as frugivorous species, some large‐bodied or canopy‐dwelling birds, and a limited number of insects capable of long‐distance pollen transport (Fahrig 2007; Greenleaf et al. 2007; Dennis et al. 2007).

Similarly, the persistence of dispersal functions within highly fragmented landscapes dominated by small patches (~30–40 ha) is unlikely to support viable populations of large‐bodied seed dispersers, including primates and terrestrial mammals, leading to a functional filtering of dispersal agents (Offerman et al. 1995; Julliot 1996; Lees and Peres 2009; Cramer et al. 2007). As a result, even where structural connectivity and stepping‐stones remain, dispersal and pollination processes are expected to become increasingly biased toward small‐seeded, generalist plant species, with important consequences for forest regeneration and long‐term compositional stability (Dennis et al. 2007; Terborgh et al. 2008; Winfree et al. 2009).

Despite these alarming trends, our results also indicate a latent potential for recovery. In some regions, inter‐fragment distances remain within the dispersal thresholds of certain plants and animals, allowing occasional movement across the landscape (Paquet et al. 2006). Even rare long‐distance dispersal events can have a disproportionate influence on population persistence and range expansion (Hardesty 2007; Higgins and Richardson 1999). Moreover, behavioral plasticity among frugivores and pollinators may partially mitigate fragmentation effects when stepping‐stones are strategically distributed (Ramos et al. 2020; Ferreira et al. 2020).

Beyond regional biodiversity impacts, the collapse of functional connectivity in the Amazon biome poses broader risks to global ecological stability. Amazon forests play a central role in climate regulation through carbon storage and water recycling (Yao et al. 2024). Fragmentation‐driven losses of ecological integrity may compromise these functions, intensifying carbon emissions, altering hydrological cycles, and increasing the likelihood of large‐scale tipping points in the Earth system (Lovejoy and Nobre 2019; Artaxo 2023; Giammarese et al. 2024).

Although the spatial patterns documented here are not unique to the Amazon forests, our results have broader implications for fragmented forest biomes worldwide. Increases in fragmentation have been reported in other tropical, subtropical, and temperate forest systems (Sodhi et al. 2004; Saura et al. 2011; Haddad et al. 2015). However, the Amazon differs critically in both scale and pace. The erosion of functional connectivity documented here has occurred over an exceptionally short period (~40 years) across vast continuous forest landscapes that, until recently, supported biome‐wide dispersal processes.

Unlike biomes such as the Brazilian Atlantic Forest, where fragmentation unfolded over centuries, Amazon ecosystems are experiencing rapid connectivity collapse with limited opportunity for ecological or evolutionary compensation (Kimmel et al. 2008; Ribeiro et al. 2009). Moreover, the strong dependence of Amazon forests on biotic dispersal and pollination, coupled with their central role in regional and global climate regulation, implies that comparable levels of fragmentation may generate disproportionately larger ecological and systemic consequences (Werth and Avissar 2002; Terborgh et al. 2002; Gardner et al. 2010; Ruiz‐Vázquez et al. 2020). Thus, while the structural dynamics of fragmentation may be generalizable, the functional and climatic implications of connectivity loss among Amazon forests are likely to be uniquely severe.

From a conservation perspective, these findings emphasize that protecting forest remnants alone is insufficient. Restoration strategies aimed at enhancing functional connectivity, such as ecological corridors, stepping‐stone networks, and assisted natural regeneration, are essential to prevent irreversible ecological degradation (Brancalion et al. 2019). Spatial planning should prioritize regions where residual connectivity persists while targeting highly fragmented areas for restoration interventions.

Lastly, current environmental governance in the Brazilian Amazon often overlooks the importance of connectivity in protected area design and infrastructure planning (Castro et al. 2020). Integrating connectivity considerations into land‐use zoning, agricultural strategies, and forest code enforcement could strengthen ecological networks and delay or prevent functional isolation (Keeley et al. 2022).

Our results allow a more explicit translation of changes in structural and functional connectivity into concrete ecological consequences for dispersal‐related processes. The sharp decline in connectivity under gap‐crossing (~310 m) and seed‐dispersal (~490 m) thresholds indicates that only a restricted subset of Amazonian organisms is likely to sustain movement among fragments under current landscape configurations. Gap distances approaching or exceeding 150–300 m are beyond the routine movement capacity of most forest‐dependent understory birds, small mammals, and many insect taxa that actively avoid open matrices, implying that connectivity at these scales is increasingly maintained by highly mobile generalists, such as some canopy frugivorous birds, bats, and large‐bodied insects capable of long‐distance pollen transport (Offerman et al. 1995; Lees and Peres 2009). Similarly, the persistence of connectivity within landscapes dominated by small fragments suggests strong functional filtering, where large‐bodied vertebrate seed dispersers are unlikely to persist or move effectively among patches (Michalski and Peres 2007; Parry et al. 2007). As a result, even where stepping‐stones remain, dispersal processes are expected to become biased toward small‐seeded plant species and generalist mutualisms, potentially altering regeneration pathways, plant community composition, and long‐term forest structure (Terborgh et al. 2008; Galetti et al. 2013). Thus, the observed erosion of functional connectivity represents not only a quantitative loss of links among patches, but also a qualitative reorganization of dispersal networks, with disproportionate consequences for species that underpin key ecosystem functions.

Forest fragmentation in the Amazon forests is closely intertwined with degradation processes such as edge effects, fire intrusion, and selective logging (Laurance et al. 2002; Cochrane and Laurance 2002; Asner et al. 2005). These processes disproportionately affect smaller and more isolated fragments, amplifying ecological deterioration beyond what is captured by forest cover loss alone. Therefore, the observed reductions in patch area and increases in isolation likely reflect not only structural fragmentation but also underlying degradation dynamics that further compromise functional connectivity.

The patterns observed here are strongly linked to ongoing land‐use changes in the Amazon Arc of Deforestation, particularly the expansion of large‐scale monocultures such as soy (Fearnside 2001). These dynamics often involve indirect land‐use displacement, whereby pasture and agricultural expansion push deforestation into remaining forest areas, accelerating fragmentation and connectivity loss (Barona et al. 2010; Arima et al. 2011). Such processes provide an important socio‐economic context for understanding the rapid structural changes observed in this study.

Beyond individual dispersal mechanisms, the combined decline in connectivity and increase in spatial modularity reveal important insights into long‐term fragmentation dynamics in the Amazon Arc of Deforestation. The progressive compartmentalization of landscapes into semi‐isolated modules suggests that ecological processes may persist locally but are increasingly decoupled at broader spatial scales, reducing landscape‐level resilience (Haddad et al. 2015; With 2019). This pattern is particularly concerning given that functional connectivity in the Amazon does not operate in isolation, but likely interacts synergistically with other drivers of biodiversity loss, such as defaunation and climate change (Berenguer et al. 2024). Reduced animal movement limits not only dispersal but also demographic and genetic rescue, amplifying the impacts of hunting, population declines, and extreme climatic events within isolated modules (Fahrig 2003; Fahrig et al. 2022). In this context, regions such as Rondônia and Eastern Pará appear to be approaching a functional fragmentation regime where recovery through natural dispersal becomes increasingly unlikely, even if deforestation rates stabilize. Conversely, the relatively higher connectivity and lower modularity observed in Western Pará highlight landscapes where conservation and restoration actions could still prevent irreversible functional isolation.

From a conservation perspective, our findings emphasize that maintaining forest cover alone is insufficient: safeguarding Amazon biodiversity and ecosystem services requires preserving and restoring the spatial conditions that allow dispersal processes to operate across realistic biological scales. By explicitly linking empirically grounded dispersal thresholds to long‐term changes in landscape reconfiguration, this study provides a framework for identifying where functional connectivity is being lost, which ecological processes are most at risk, and where targeted interventions may still yield disproportionate ecological benefits.

## Author Contributions

**Mario Arthur Favretto:** conceptualization, investigation, writing – original draft, methodology, writing – review and editing, visualization, formal analysis. **Marina Hirota:** conceptualization, investigation, writing – original draft, methodology, visualization, writing – review and editing, formal analysis, supervision.

## Conflicts of Interest

The authors declare no conflicts of interest.

## Supporting information


**Table S1:** Data used to determine the ecological distance thresholds in Amazon forest, with species, distance, ecological threshold and reference.

**Data S1:** gcb70959‐sup‐0002‐Supinfo02.rar.

## Data Availability

The supplemental material of this study is openly available in Zenodo at https://doi.org/10.5281/zenodo.20444498.

## References

[gcb70959-bib-0001] Albert, J. S. , A. C. Carnaval , S. G. A. Flantua , et al. 2023. “Human Impacts Outpace Natural Processes in the Amazon.” Science 379: 348. 10.1126/science.abo5003. 36701466

[gcb70959-bib-0002] Arima, E. Y. , P. Richards , R. Walker , and M. M. Caldas . 2011. “Statistical Confirmation of Indirect Land Use Change in the Brazilian Amazon.” Enviromental Research Letters 6: 024010.

[gcb70959-bib-0003] Artaxo, P. 2023. “Amazon Deforestation Implications in Local/Regional Climate Change.” Proceedings of the National Academy of Sciences 120: e2317456120. 10.1073/pnas.2317456120. PMC10722968 38032950

[gcb70959-bib-0004] Asner, G. P. , D. E. Knapp , E. N. Broadbent , P. J. C. Oliveira , M. Keller , and J. N. Silva . 2005. “Selective Logging in the Brazilian Amazon.” Science 310: 480–482. 10.1126/science.1118051. 16239474

[gcb70959-bib-0005] Barona, E. , N. Ramankutty , G. Hyman , and O. T. Coomes . 2010. “The Role of Pasture and Soybean in Deforestation of the Brazilian Amazon.” Enviromental Research Letters 5: 024002.

[gcb70959-bib-0006] Berenguer, E. , D. Armenteras , A. C. Lees , et al. 2024. “Drivers and Ecological Impacts of Deforestation and Forest Degradation in the Amazon.” Acta Amazonica 54: e54es22342. 10.1590/1809-4392202203420.

[gcb70959-bib-0007] Brancalion, P. H. S. , A. Niamir , E. Broadbent , et al. 2019. “Global Restoration Opportunities in Tropical Rainforest Landscapes.” Science Advances 5: eaav3223. 10.1126/sciadv.aav3223. 31281881 PMC6609219

[gcb70959-bib-0008] Brandes, U. , D. Delling , M. Gaertler , et al. 2008. “On Modularity Clustering.” IEEE Transactions of Knowledge and Data Engineering 20: 172–188.

[gcb70959-bib-0009] Castro, R. B. , J. L. G. Pereira , M. Zanin , and A. L. K. M. Albernaz . 2020. “Connectivity, Spatial Structure and the Identification of Priority Areas for Conservation of Belém Area of Endemism, Amazon.” Anais da Academia Brasileira de Ciências 92: e20181357. 10.1590/0001-3765202020181357. 32785424

[gcb70959-bib-0010] Claramunt, S. , M. Hong , and A. Bravo . 2022. “The Effect of Flight Efficiency on Gap‐Crossing Ability in Amazonian Forest Birds.” Biotropica 54: 860–868. 10.1111/btp.13109.

[gcb70959-bib-0011] Cochrane, M. A. , and W. F. Laurance . 2002. “Fire as a Large‐Scale Edge Effect in Amazonian Forests.” Journal of Tropical Ecology 18: 311–325. 10.1017/S0266467402002237.

[gcb70959-bib-0012] Collinge, S. K. 2009. Ecology of Fragmented Landscapes. Johns Hopkins University Press.

[gcb70959-bib-0013] Cramer, J. M. , R. C. G. Mesquita , and G. B. Williamson . 2007. “Forest Fragmentation Differentially Affects Seed Dispersal of Large and Small‐Seeded Tropical Trees.” Biological Conservation 137: 415–423. 10.1016/j.biocon.2007.02.019.

[gcb70959-bib-0014] Cui, J. , S. Piao , C. Huntingford , T. Wang , and D. V. Spracklen . 2026. “Historical Deforestation Drives Strong Rainfall Decline Across the Southern Amazon Basin.” Nature Communications 17: 1642. 10.1038/s41467-026-68361-z. PMC12905307 41530197

[gcb70959-bib-0015] Cumming, G. S. 2011. Spatial Resilience in Social‐Ecological Systems. Springer.

[gcb70959-bib-0016] Dennis, A. J. , E. W. Schupp , R. J. Green , and D. A. Westcott . 2007. “Seed Dispersal: Theory and Its Application in a Changing World.” CAB International, UK.

[gcb70959-bib-0017] Didham, R. K. , V. Kapos , and R. M. Ewers . 2012. “Rethinking the Conceptual Foundations of Habitat Fragmentation Research.” Oikos 121: 161–170. 10.1111/j.1600-0706.2011.20273.x.

[gcb70959-bib-0018] Du, Y. , G. Gao , X. Ma , S. Xu , and B. Fu . 2025. “Amazon Basin Shows Reduced Forest Loss but Increased Forest Spatial Fragmentation in 1992–2020.” Science of the Total Environment 990: 179917. 10.1016/j.scitotenv.2025.179917. 40554158

[gcb70959-bib-0019] Duane, A. , M. D. Miranda , and L. Brotons . 2021. “Forest Connectivity Percolation Thresholds for Fire Spread Under Different Weather Conditions.” Forest Ecology and Management 498: 119558.

[gcb70959-bib-0020] Emer, C. , M. Galetti , M. A. Pizo , et al. 2018. “Seed‐Dispersal Interactions in Fragmented Landscapes ‐ a Metanetwork Approach.” Ecology Letters 21: 484–493. 10.1111/ele.12909. 29368364

[gcb70959-bib-0021] Fahrig, L. 2003. “Effects of Habitat Fragmentation on Biodiversity.” Annual Review of Ecology, Evolution, and Systematics 34: 487–515. 10.1146/annurev.ecolsys.34.011802.132419.

[gcb70959-bib-0022] Fahrig, L. 2007. “Non‐Optimal Animal Movement in Human‐Altered Landscapes.” Functional Ecology 21: 1003–1015. 10.1111/j.1365-2435.2007.01326.x.

[gcb70959-bib-0023] Fahrig, L. , V. Arroyo‐Rodríguez , E. Cazetta , A. Ford , J. Lancaster , and T. Ranius . 2022. “Landscape Connectivity.” In The Routledge Handbook of Landscape Ecology, edited by R. A. Francis , J. D. A. Millington , G. L. W. Perry , and E. S. Minor , 67–88. Taylor and Francis Group.

[gcb70959-bib-0024] Favretto, M. A. , and M. Hirota . 2026. “Connectivity Structure in Fragmented Forest Areas in the Brazilian Amazon.” Biological Conservation 316: 111747. 10.1016/j.biocon.2026.111747.

[gcb70959-bib-0025] Fearnside, P. M. 2001. “Soybean Cultivation as a Threat to the Environment in Brazil.” Environmental Conservation 28, no. 1: 23–38. 10.1017/S0376892901000030.

[gcb70959-bib-0026] Fearnside, P. M. 2005. “Deforestation in Brazilian Amazonia: History, Rates, and Consequences.” Conservation Biology 19: 680–688. 10.1111/j.1523-1739.2005.00697.x.

[gcb70959-bib-0027] Fearnside, P. M. 2021. “Desmatamento na Amazônia Brasileira: história, índices e consequências.” In Destruição e conservação da Floresta Amazônica, edited by P. M. Fearnside , 7–19. INPA.

[gcb70959-bib-0028] Ferraz, G. , G. J. Russell , P. C. Stouffer , R. O. Bierregaard , S. L. Pimm , and T. E. Lovejoy . 2003. “Rates of Species Loss From Amazonian Forest Fragments.” Proceedings of the National Academy of Sciences 100: 14069–14073. 10.1073/pnas.2336195100. PMC283547 14614134

[gcb70959-bib-0029] Ferreira, P. A. , D. Boscolo , L. E. Lopes , et al. 2020. “Forest and Connectivity Loss Simplify Tropical Pollination Networks.” Oecologia 192: 577–590. 10.1007/s00442-019-04579-7. 31897723

[gcb70959-bib-0030] Foltête, J. C. , G. Vuidel , P. Savary , et al. 2021. “Graphab: An Application for Modeling and Managing Ecological Habitat Networks.” Software Impacts 8: 100065.

[gcb70959-bib-0031] Franco, M. A. , L. V. Rizzo , M. J. Teixeira , et al. 2025. “How Climate Change and Deforestation Interact in the Transformation of the Amazon Rainforest.” Nature Communications 16: 7944. 10.1038/s41467-025-63156-0. PMC12405584 40897684

[gcb70959-bib-0032] Galán‐Acedo, C. , R. Arasa‐Gisbert , V. Arroyo‐Rodríguez , M. Martínez‐Ruiz , F. A. Rosete‐Vergés , and F. Villalobos . 2023. “Effects of Habitat Loss on Brazilian Primates: Assessing Extinction Thresholds in the Amazon and Atlantic Forest.” Perspectives in Ecology and Conservation 21: 189–195. 10.1016/j.pecon.2023.05.001.

[gcb70959-bib-0033] Galetti, M. , R. Guevara , M. C. Côrtes , et al. 2013. “Functional Extinction of Birds Drives Rapid Evolutionary Changes in Seed Size.” Science 304: 1086–1090. 10.1126/science.1233774. 23723235

[gcb70959-bib-0034] Gardener, M. 2017. Statistics for Ecologists Using R and Excel: Data Collection, Exploration, Analysis and Presentation. Pelagic Publishing.

[gcb70959-bib-0035] Gardner, T. A. , J. Barlow , N. S. Sodhi , and C. A. Peres . 2010. “A Multi‐Region Assessment of Tropical Forest Biodiversity in a Human‐Modified World.” Biological Conservation 143: 2293–2300. 10.1016/j.biocon.2010.05.017.

[gcb70959-bib-0036] Gatti, L. V. , C. L. Cunha , L. Marani , et al. 2023. “Increased Amazon Carbon Emissions Mainly From Decline in Law Enforcement.” Nature 621: 318–323. 10.1038/s41586-023-06390-0. 37612502

[gcb70959-bib-0037] Giammarese, A. , J. Brown , and N. Malik . 2024. “Reconfiguration of Amazon's Connectivity in the Climate System.” Chaos 34: 013134. 10.1063/5.0165861. 38260937

[gcb70959-bib-0038] Greenleaf, S. S. , N. M. Williams , R. Winfree , and C. Kremen . 2007. “Bee Foraging Ranges and Their Relationship to Body Size.” Plant Animal Interactions 153: 589–596. 10.1007/s00442-007-0752-9. 17483965

[gcb70959-bib-0039] Haddad, N. M. , L. A. Brudvig , J. Clobert , et al. 2015. “Habitat Fragmentation and Its Lasting Impact on Earth's Ecosystems.” Science Advances 1: e1500052. 10.1126/sciadv.1500052. 26601154 PMC4643828

[gcb70959-bib-0040] Hanski, I. 2011. “Habitat Loss, the Dynamics of Biodiversity, and a Perspective on Conservation.” Ambio 40: 248–255. 10.1007/s13280-011-0147-3. 21644453 PMC3357798

[gcb70959-bib-0041] Hardesty, B. D. 2007. “How Far Do Offspring Recruit From Parent Plants? A Molecular Approach to Understanding Effective Dispersal.” In Seed Dispersal: Theory and Its Application in a Changing World, edited by A. J. Dennis , E. W. Schupp , R. J. Green , and D. A. Westcott , 277–299. CABI.

[gcb70959-bib-0042] He, J. , J. Huang , D. Liu , H. Wang , and C. Li . 2018. “Updating the Habitat Conservation Institution by Prioritizing Important Connectivity and Resilience Providers Outside.” Ecological Indicators 88: 219–231. 10.1016/j.ecolind.2017.12.067.

[gcb70959-bib-0043] Higgins, S. I. , and D. M. Richardson . 1999. “Predicting Plant Migration Rates in a Changing World: The Role of Long‐Distance Dispersal.” American Naturalist 153: 464–475. 10.1086/303193. 29578791

[gcb70959-bib-0044] Hooper, E. R. , and M. S. Ashton . 2020. “Fragmentation Reduces Community‐Wide Taxonomic and Functional Diversity of Dispersed Tree Seeds in the Central Amazon.” Ecological Applications 30: e02093. 10.1002/eap.2093. 32065685

[gcb70959-bib-0045] Imong, I. , M. M. Robbins , R. Mundry , R. Bergl , and H. S. Kühl . 2014. “Informing Conservation Management About Structural Versus Functional Connectivity: A Case‐Study of Cross River Gorillas.” American Journal of Primatology 76: 978–988. 10.1002/ajp.22287. 24737604

[gcb70959-bib-0046] Issii, T. M. , E. F. L. Pereira‐Silva , C. T. L. Pablo , R. F. Santos , and E. Hardt . 2020. “Is There an Equivalence Between Measures of Landscape Structural and Functional Connectivity for Plants in Conservation Assessments of the Cerrado?” Land 9: 459. 10.3390/land9110459.

[gcb70959-bib-0047] JASP Team . 2023. “JASP (Version 0.17)[Computer Software].”

[gcb70959-bib-0048] Julliot, C. 1996. “Seed Dispersal by Red Howling Monkeys ( *Alouatta seniculus* ) in the Tropical Rain Forest of French Guiana.” International Journal of Primatology 17: 239–258. 10.1007/BF02735451. 31918515

[gcb70959-bib-0049] Keeley, A. T. H. , A. K. Fremier , P. A. L. Goertler , et al. 2022. “Governing Ecological Connectivity in Cross‐Scale Dependent Systems.” Bioscience 72: 372–386. 10.1093/biosci/biab140. 35370478 PMC8970826

[gcb70959-bib-0050] Kimmel, T. , D. Piechowski , and G. Gottsberger . 2008. “The History of Fragmentation of the Lowland Atlantic Forest of Pernambuco, Brazil.” Bioremediation, Biodiversity and Bioavailability 2: 1–4.

[gcb70959-bib-0051] Laurance, W. F. , M. A. Cochrane , S. Bergen , et al. 2001. “The Future of the Brazilian Amazon.” Science 291: 438–439. 10.1126/science.291.5503.438. 11228139

[gcb70959-bib-0052] Laurance, W. F. , P. Delamônica , S. G. Laurance , H. L. Vasconcelos , and T. E. Lovejoy . 2000. “Rain Forest Fragmentation Kills Big Trees.” Nature 404: 836. 10.1038/35009032. 10786782

[gcb70959-bib-0053] Laurance, W. F. , T. E. Lovejoy , H. L. Vasconcelos , et al. 2002. “Ecosystem Decay of Amazonian Forest Fragments: A 22‐Year Investigation.” Conservation Biology 16: 605–618. 10.1046/j.1523-1739.2002.01025.x.

[gcb70959-bib-0054] Lees, A. C. , and C. A. Peres . 2009. “Gap‐Crossing Movements Predict Species Occupancy in Amazonian Forest Fragments.” Oikos 118: 280–290. 10.1111/j.1600-0706.2008.16842.x.

[gcb70959-bib-0055] Liu, X. , D. Li , B. K. Szymanski , H. E. Stanley , and J. Gao . 2022. “Network Resilience.” Physics Reports 971: 1–108. 10.1016/j.physrep.2022.04.002.

[gcb70959-bib-0056] Lovejoy, T. E. , and C. Nobre . 2019. “Amazon Tipping Point: Last Chance for Action.” Science Advances 5: eaba2949. 10.1126/sciadv.aba2949. 32064324 PMC6989302

[gcb70959-bib-0057] MapBiomas . 2022. “MapBiomas.” https://mapbiomas.org/.

[gcb70959-bib-0058] Matricardi, E. A. T. , D. L. Skole , O. B. Costa , M. A. Pedlowski , J. H. Samek , and E. P. Miguel . 2020. “Long‐Term Forest Degradation Surpasses Deforestation in the Brazilian Amazon.” Science 369: 1378–1382. 10.1126/science.abb3021. 32913104

[gcb70959-bib-0059] McGarigal, K. , S. A. Cushman , and E. Ene . 2012. “FRAGSTATS v4: Spatial Pattern Analysis Program for Categorical and Continuous Maps.” Amherst, MA: Computer Software Program Produced by the Authors at the University of Massachusetts. http://www.umass.edu/landeco/research/fragstats/fragstats.html.

[gcb70959-bib-0060] Michalski, F. , and C. A. Peres . 2007. “Disturbance‐Mediated Mammal Persistence and Abundance‐Area Relationships in Amazonian Forest Fragments.” Conservation Biology 21: 1626–1640. 10.1111/j.1523-1739.2007.00797.x. 18173486

[gcb70959-bib-0061] Miranda, L. S. , M. Awade , R. Jaffé , et al. 2021. “Combining Connectivity and Species Distribution Modeling to Define Conservation and Restoration Priorities for Multiple Species: A Case Study in the Eastern Amazon.” Biological Conservation 257: 109148. 10.1016/j.biocon.2021.109148.

[gcb70959-bib-0062] Murcia, C. 1995. “Edge Effects in Fragmented Forests: Implications for Conservation.” Trends in Ecology & Evolution 10: 58–62. 10.1016/S0169-5347(00)88977-6. 21236953

[gcb70959-bib-0063] Newman, M. E. J. 2006. “Modularity and Community Structure in Networks.” Proceedings of the National Academy of Sciences 103: 8577–8582. 10.1073/pnas.0601602103. PMC1482622 16723398

[gcb70959-bib-0064] Nobre, C. A. , G. Sampaio , L. S. Borma , J. C. Castilla‐Rubio , J. S. Silva , and M. Cardoso . 2016. “Land‐Use and Climate Change Risks in the Amazon and the Need of a Novel Sustainable Development Paradigm.” Proceedings of the National Academy of Sciences 113: 10759–10768. 10.1073/pnas.1605516113. PMC5047175 27638214

[gcb70959-bib-0065] Offerman, H. L. , V. H. Dale , S. M. Pearson , R. O. Bierregaard , and R. V. O'Neill . 1995. “Effects of Forest Fragmentation on Neotropical Fauna: Current Research and Data Availability.” Environmental Review 3: 191–211. 10.1139/a95-009.

[gcb70959-bib-0066] Paquet, P. C. , S. M. Alexander , P. L. Swan , and C. T. Darimont . 2006. “Influence of Natural Landscape Fragmentation and Resource Availability on Distribution and Connectivity of Gray Wolves ( *Canis lupus* ) in the Archipelago of Coastal British Columbia, Canada.” In Connectivity Conservation, edited by K. R. Crooks and M. Sanjayan , 130–156. Cambridge University Press.

[gcb70959-bib-0067] Parry, L. , J. Barlow , and C. A. Peres . 2007. “Large‐Vertebrate Assemblages of Primary and Secondary Forests in the Brazilian Amazon.” Journal of Tropical Ecology 23: 653–662. 10.1017/S0266467407004506.

[gcb70959-bib-0068] Passel, J. V. , P. M. Bernardino , S. Lhermitte , et al. 2024. “Critical Slowing Down of the Amazon Forest After Increased Drought Occurrence.” Proceedings of the National Academy of Sciences 121: e2316924121. 10.1073/pnas.2316924121. PMC11145287 38768350

[gcb70959-bib-0069] Ramos, D. L. , M. A. Pizo , M. C. Ribeiro , R. S. Cruz , J. M. Morales , and O. Ovaskainen . 2020. “Forest and Connectivity Loss Drive Changes in Movement Behavior of Bird Species.” Ecography 43: 1203–1214. 10.1111/ecog.04888.

[gcb70959-bib-0070] Ribeiro, M. C. , J. P. Metzger , A. C. Martensen , F. J. Ponzoni , and M. M. Hirota . 2009. “The Brazilian Atlantic Forest: How Much Is Left, and How Is the Remaining Forest Distributed? Implications for Conservation.” Biological Conservation 142: 1141–1153. 10.1016/j.biocon.2009.02.021.

[gcb70959-bib-0071] Ritter, C. D. , J. Muñoz , A. F. Machado , et al. 2025. “Indigenous Territories and Protected Areas Are Crucial for Ecosystem Connectivity in the Amazon Basin.” Proceedings of the National Academy of Sciences 122: e2418189122. 10.1073/pnas.2418189122. PMC12337320 40720645

[gcb70959-bib-0072] Rivas, C. A. , and R. M. Navarro‐Cerrillo . 2024. “Forest Fragmentation and Connectivity in South American Dry Forests.” Biodiversity and Conservation 33: 3015–3037. 10.1007/s10531-024-02894-x.

[gcb70959-bib-0073] Ruiz‐Vázquez, M. , P. A. Arias , J. A. Martínez , and J. C. Espinoza . 2020. “Effects of Amazon Basin Deforestation on Regional Atmospheric Circulation and Water Vapor Transport Towards Tropical South America.” Climate Dynamics 54: 4169–4189. 10.1007/s00382-020-05223-4.

[gcb70959-bib-0074] Santos, R. C. , M. Lima , C. A. Silva Junior , and L. D. Battirola . 2019. “Disordered Conversion of Vegetation Committees Connectivity Between Forest Fragments in the Brazilian Legal Amazon.” Applied Geography 111: 102082. 10.1016/j.apgeog.2019.102082.

[gcb70959-bib-0075] Saura, S. , C. Estreguil , C. Mouton , and M. Rodríguez‐Freire . 2011. “Network Analysis to Assess Landscape Connectivity Trends: Application to European Forests (1990–2000).” Ecological Indicators 11: 407–416. 10.1016/j.ecolind.2010.06.011.

[gcb70959-bib-0076] Saura, S. , and B. Fuente . 2017. “Connectivity as the Amount of Reachable Habitat: Conservation Priorities and the Roles of Habitat Patches in Landscape Networks.” In Learning Landscape Ecology: A Practical Guide to Concepts and Techniques, edited by S. E. Gergel and M. G. Turner , 229–254. Springer.

[gcb70959-bib-0077] Saura, S. , and L. Pascual‐Hortal . 2007. “A New Habitat Availability Index to Integrate Connectivity in Landscape Conservation Planning: Comparison With Existing Indices and Application to a Case Study.” Landscape and Urban Planning 83: 91–103. 10.1016/j.landurbplan.2007.03.005.

[gcb70959-bib-0078] Scheffer, M. , S. R. Carpenter , T. M. Lenton , et al. 2012. “Anticipating Critical Transitions.” Science 388: 344–348. 10.1126/science.1225244. 23087241

[gcb70959-bib-0079] Siegel, T. , A. Magrach , W. F. Laurance , and D. Luther . 2024. “A Global Meta‐Analysis of the Impacts of Forest Fragmentation on Biotic Mutualisms and Antagonisms.” Conservation Biology 38: e14206. 10.1111/cobi.14206. 37855172

[gcb70959-bib-0080] Smith, M. M. , and J. N. Pauli . 2024. “Small but Connected Islands Can Maintain Populations and Genetic Diversity Under Climate Change.” Ecography 2024: e07119. 10.1111/ecog.07119.

[gcb70959-bib-0081] Sodhi, N. S. , L. P. Koh , B. W. Brook , and P. K. L. Ng . 2004. “South Asian Biodiversity: An Impending Disaster.” Trends in Ecology & Evolution 19: 654–660. 10.1016/j.tree.2004.09.006. 16701328

[gcb70959-bib-0082] Taylor, P. D. , L. Fahrig , K. Henein , and G. Merriam . 1993. “Connectivity Is a Vital Element of Landscape Structure.” Oikos 68: 571–573. 10.2307/3544927.

[gcb70959-bib-0083] Teixeira, C. , G. L. Nunes , L. C. Trevelin , D. P. Silva , and A. L. C. Prudente . 2025. “Assessing Connectivity Thresholds Under Habitat Loss Scenarios for Threatened Amphibians and Squamate Reptiles in the Eastern Brazilian Amazon.” Ecology and Evolution 15: e71741. 10.1002/ece3.71741. 40666690 PMC12260477

[gcb70959-bib-0084] Terborgh, J. , G. N. Iturri , N. C. A. Pitman , et al. 2008. “Tree Recruitment in an Empty Forest.” Ecology 89: 1757–1768. 10.1890/07-0479.1. 18589539

[gcb70959-bib-0085] Terborgh, J. , N. Pitman , M. Silman , and H. Schichter . 2002. “Maintenance of Tree Diversity in Tropical Forests.” In Seed Dispersal and Frugivory: Ecology, Evolution and Conservation, edited by D. J. Levey , W. R. Silva , and M. Galetti , 1–17. CAB International.

[gcb70959-bib-0086] Turner, M. G. , and R. H. Gardner . 2015. Landscape Ecology in Theory and Practice. Springer.

[gcb70959-bib-0087] Tylianakis, J. M. , T. A. Rand , A. Kahmen , et al. 2008. “Resource Heterogeneity Moderates the Biodiversity‐Function Relationship in Real World Ecosystems.” PLoS One 6: e122. 10.1371/journal.pbio.0060122.

[gcb70959-bib-0088] Urban, D. , and T. Keitt . 2001. “Landscape Connectivity: A Graph‐Theoretic Perspective.” Ecology 82: 1205–1218. 10.1890/0012-9658(2001)082[1205:LCAGTP]2.0.CO;2.

[gcb70959-bib-0089] Vitali, A. , S. Ruiz‐Suarez , D. P. Vázquez , et al. 2023. “Invasive Species Modulate the Structure and Stability of a Multilayer Mutualistic Network.” Proceedings of the Royal Society B 290: 20230132. 10.1098/rspb.2023.0132. 37357855 PMC10291717

[gcb70959-bib-0090] Werth, D. , and R. Avissar . 2002. “The Local and Global Effects of Amazon Deforestation.” Journal of Geophysical Research 107: 8087. 10.1029/2001JD000717.

[gcb70959-bib-0091] Winfree, R. , R. Aguilar , D. P. Vázquez , G. LeBuhn , and M. A. Aizen . 2009. “A Meta‐Analysis of Bees' Responses to Anthropogenic Disturbance.” Ecology 90: 2068–2076. 10.1890/08-1245.1. 19739369

[gcb70959-bib-0092] With, K. A. 2019. Essentials of Landscape Ecology. Oxford University Press.

[gcb70959-bib-0093] Yao, Y. , P. Ciais , E. Joetzjer , et al. 2024. “The Impacts of Elevated CO2 on Forest Growth, Mortality, and Recovery in the Amazon Rainforest.” Earth System Dynamics 15: 763–778. 10.5194/esd-15-763-2024.

[gcb70959-bib-0094] Yoshikawa, S. , and K. Sanga‐Ngoie . 2011. “Deforestation Dynamics in Mato Grosso in the Southern Brazilian Amazon Using GIS and NOAA/AVHRR Data.” International Journal of Remote Sensing 32: 523–544. 10.1080/01431160903475225.

