# Supplementary material for: Rapid and Intense Declines of Forest Connectivity in the Amazon Arc of Deforestation Over Four Decades
Source: Glob Chang Biol. 2026 Jun 6;32(6):e70959. doi: 10.1111/gcb.70959 (PMC13241863; doi:10.1111/gcb.70959)
Supplement: Supplementary file 1 — Table S1: Data used to determine the ecological distance thresholds in Amazon forest, with species, distance, ecological threshold and reference. [file GCB-32-e70959-s002.docx]

**Defining distance thresholds**

**Literature review**

To define distance thresholds, we use the methodology of Favretto & Hirota (unpublished data).

We conducted a targeted narrative literature review with systematic elements, aiming to compile empirical estimates of dispersal and movement distances relevant to functional connectivity in Amazonian forests. Although not a formal systematic review or meta-analysis, our approach followed predefined search strings, databases, and inclusion criteria, focusing exclusively on studies conducted within Amazonian forest landscapes.

We targeted three main ecological processes that facilitate connectivity: (1) seed dispersal distances; (2) pollen dispersal distances; and (3) the gap-crossing capacities of various animal species that move between forest fragments across anthropogenic matrices. Gap-crossing is treated here not as an ecological process per se, but as a functional movement capacity that underpins multiple ecological processes, including seed dispersal, pollination, gene flow, and demographic rescue, particularly in fragmented landscapes. We restricted our search to studies conducted specifically in Amazonian forests, given the high dependence of most species on continuous forest cover and sensitivity to deforestation.

Here, we define functional distance thresholds as empirically derived reference distances synthesised from published studies that quantify the maximum or effective distances over which organisms are able to move or disperse between habitat patches. These thresholds do not represent absolute physiological limits, but rather operational parameters that reflect the spatial scale at which ecological processes are likely to remain functional in fragmented Amazonian landscapes.

Searches were conducted in Google Scholar, Scopus, SciELO and ResearchGate using Boolean operators and wildcard symbols, combining terms related to dispersal, connectivity and fragmentation, i.e. (Amazon OR "Amazon forest" OR Amazonia) AND (dispersal OR "seed dispersal" OR "plant dispersal" OR "pollen dispersal" OR "insect dispersal") AND (connectivity OR "forest connectivity" OR "fragment isolation" OR "forest fragments" OR "gap crossing") AND (plants OR insects OR birds OR amphibians) AND (distance OR "dispersal distance" OR "distance between forest fragments"). Studies were included if they (i) reported empirical distance estimates, (ii) were conducted in Amazonian forests, and (iii) explicitly addressed movement or dispersal across habitat gaps. After the searches, 28 references were selected and used for the present study (Suppl. Table 1).

Supplementary table 1. Data used to determine the ecological distance thresholds in Amazon forest, with species, distance, ecological threshold and reference.

| **Group** | **Species** | **Maximum Dispersal Distance (m)** | **Ecological threshold** | **Reference** |
| --- | --- | --- | --- | --- |
| Bird | *Thamnophilus aethiops* | 7 | Gap-crossing | Lees, A.C., Peres, C.A., 2009. Gap-crossing movements predict species occupancy in Amazonian forest fragments. Oikos 118, 280-290. https://doi.org/10.1111/j.1600-0706.2008.16842.x |
| Bird | *Popelairia langsdorffi* | 7 | Gap-crossing | Lees, A.C., Peres, C.A., 2009. Gap-crossing movements predict species occupancy in Amazonian forest fragments. Oikos 118, 280-290. https://doi.org/10.1111/j.1600-0706.2008.16842.x |
| Bird | *Sclerurus albigularis* | 8 | Gap-crossing | Lees, A.C., Peres, C.A., 2009. Gap-crossing movements predict species occupancy in Amazonian forest fragments. Oikos 118, 280-290. https://doi.org/10.1111/j.1600-0706.2008.16842.x |
| Bird | *Touit huettii* | 9 | Gap-crossing | Lees, A.C., Peres, C.A., 2009. Gap-crossing movements predict species occupancy in Amazonian forest fragments. Oikos 118, 280-290. https://doi.org/10.1111/j.1600-0706.2008.16842.x |
| Bird | *Xiphorhynchus guttatus* | 12 | Gap-crossing | Lees, A.C., Peres, C.A., 2009. Gap-crossing movements predict species occupancy in Amazonian forest fragments. Oikos 118, 280-290. https://doi.org/10.1111/j.1600-0706.2008.16842.x |
| Bird | *Trogon viridis* | 12 | Gap-crossing | Lees, A.C., Peres, C.A., 2009. Gap-crossing movements predict species occupancy in Amazonian forest fragments. Oikos 118, 280-290. https://doi.org/10.1111/j.1600-0706.2008.16842.x |
| Bird | *Dendrocolaptes picumnus* | 12 | Gap-crossing | Lees, A.C., Peres, C.A., 2009. Gap-crossing movements predict species occupancy in Amazonian forest fragments. Oikos 118, 280-290. https://doi.org/10.1111/j.1600-0706.2008.16842.x |
| Beetle | *Canthon triangularis* | 15 | Gap-crossing | Offerman, H.L., Dale, V.H., Pearson, S.M., Bierregaard, R.O., O'Neill, R.V., 1995. Effects of forest fragmentation on neotropical fauna: current research and data availability. Environ. Rev. 3, 191-211. https://doi.org/10.1139/a95-009 |
| Beetle | *Deltochilum guyanensis* | 15 | Gap-crossing | Offerman, H.L., Dale, V.H., Pearson, S.M., Bierregaard, R.O., O'Neill, R.V., 1995. Effects of forest fragmentation on neotropical fauna: current research and data availability. Environ. Rev. 3, 191-211. https://doi.org/10.1139/a95-009 |
| Beetle | *Deltochilum septemstriatum* | 15 | Gap-crossing | Offerman, H.L., Dale, V.H., Pearson, S.M., Bierregaard, R.O., O'Neill, R.V., 1995. Effects of forest fragmentation on neotropical fauna: current research and data availability. Environ. Rev. 3, 191-211. https://doi.org/10.1139/a95-009 |
| Beetle | *Eurysternus caribaeus* | 15 | Gap-crossing | Offerman, H.L., Dale, V.H., Pearson, S.M., Bierregaard, R.O., O'Neill, R.V., 1995. Effects of forest fragmentation on neotropical fauna: current research and data availability. Environ. Rev. 3, 191-211. https://doi.org/10.1139/a95-009 |
| Beetle | *Eurysternus velutinus* | 15 | Gap-crossing | Offerman, H.L., Dale, V.H., Pearson, S.M., Bierregaard, R.O., O'Neill, R.V., 1995. Effects of forest fragmentation on neotropical fauna: current research and data availability. Environ. Rev. 3, 191-211. https://doi.org/10.1139/a95-009 |
| Bird | *Piaya cayana* | 75 | Gap-crossing | Lees, A.C., Peres, C.A., 2009. Gap-crossing movements predict species occupancy in Amazonian forest fragments. Oikos 118, 280-290. https://doi.org/10.1111/j.1600-0706.2008.16842.x |
| Bird | *Campylorhynchus turdinus* | 75 | Gap-crossing | Lees, A.C., Peres, C.A., 2009. Gap-crossing movements predict species occupancy in Amazonian forest fragments. Oikos 118, 280-290. https://doi.org/10.1111/j.1600-0706.2008.16842.x |
| Bee | *Euglossa chalybeata* | 100 | Gap-crossing | Offerman, H.L., Dale, V.H., Pearson, S.M., Bierregaard, R.O., O'Neill, R.V., 1995. Effects of forest fragmentation on neotropical fauna: current research and data availability. Environ. Rev. 3, 191-211. https://doi.org/10.1139/a95-009 |
| Bee | *Euglossa crassipunctata* | 100 | Gap-crossing | Offerman, H.L., Dale, V.H., Pearson, S.M., Bierregaard, R.O., O'Neill, R.V., 1995. Effects of forest fragmentation on neotropical fauna: current research and data availability. Environ. Rev. 3, 191-211. https://doi.org/10.1139/a95-009 |
| Bee | *Euglossa iopyrrha* | 100 | Gap-crossing | Offerman, H.L., Dale, V.H., Pearson, S.M., Bierregaard, R.O., O'Neill, R.V., 1995. Effects of forest fragmentation on neotropical fauna: current research and data availability. Environ. Rev. 3, 191-211. https://doi.org/10.1139/a95-009 |
| Bee | *Euglossa stilbonota* | 100 | Gap-crossing | Offerman, H.L., Dale, V.H., Pearson, S.M., Bierregaard, R.O., O'Neill, R.V., 1995. Effects of forest fragmentation on neotropical fauna: current research and data availability. Environ. Rev. 3, 191-211. https://doi.org/10.1139/a95-009 |
| Monkey | *Sanguinus midas* | 150 | Gap-crossing | Offerman, H.L., Dale, V.H., Pearson, S.M., Bierregaard, R.O., O'Neill, R.V., 1995. Effects of forest fragmentation on neotropical fauna: current research and data availability. Environ. Rev. 3, 191-211. https://doi.org/10.1139/a95-009 |
| Bird | *Dendrocincla merula* | 200 | Gap-crossing | Offerman, H.L., Dale, V.H., Pearson, S.M., Bierregaard, R.O., O'Neill, R.V., 1995. Effects of forest fragmentation on neotropical fauna: current research and data availability. Environ. Rev. 3, 191-211. https://doi.org/10.1139/a95-009 |
| Bird | *Dendrocolaptes certhia* | 200 | Gap-crossing | Offerman, H.L., Dale, V.H., Pearson, S.M., Bierregaard, R.O., O'Neill, R.V., 1995. Effects of forest fragmentation on neotropical fauna: current research and data availability. Environ. Rev. 3, 191-211. https://doi.org/10.1139/a95-009 |
| Ant | *Eciton burchelli* | 250 | Gap-crossing | Offerman, H.L., Dale, V.H., Pearson, S.M., Bierregaard, R.O., O'Neill, R.V., 1995. Effects of forest fragmentation on neotropical fauna: current research and data availability. Environ. Rev. 3, 191-211. https://doi.org/10.1139/a95-009 |
| Bird | *Deconychura longicauda* | 250 | Gap-crossing | Offerman, H.L., Dale, V.H., Pearson, S.M., Bierregaard, R.O., O'Neill, R.V., 1995. Effects of forest fragmentation on neotropical fauna: current research and data availability. Environ. Rev. 3, 191-211. https://doi.org/10.1139/a95-009 |
| Bird | *Xiphorhynchus pardalotus* | 250 | Gap-crossing | Offerman, H.L., Dale, V.H., Pearson, S.M., Bierregaard, R.O., O'Neill, R.V., 1995. Effects of forest fragmentation on neotropical fauna: current research and data availability. Environ. Rev. 3, 191-211. https://doi.org/10.1139/a95-009 |
| Bird | *Willisornis poecilonotus* | 250 | Gap-crossing | Offerman, H.L., Dale, V.H., Pearson, S.M., Bierregaard, R.O., O'Neill, R.V., 1995. Effects of forest fragmentation on neotropical fauna: current research and data availability. Environ. Rev. 3, 191-211. https://doi.org/10.1139/a95-009 |
| Bird | *Myrmotherula axillaris* | 250 | Gap-crossing | Offerman, H.L., Dale, V.H., Pearson, S.M., Bierregaard, R.O., O'Neill, R.V., 1995. Effects of forest fragmentation on neotropical fauna: current research and data availability. Environ. Rev. 3, 191-211. https://doi.org/10.1139/a95-009 |
| Bird | *Microbates collaris* | 250 | Gap-crossing | Offerman, H.L., Dale, V.H., Pearson, S.M., Bierregaard, R.O., O'Neill, R.V., 1995. Effects of forest fragmentation on neotropical fauna: current research and data availability. Environ. Rev. 3, 191-211. https://doi.org/10.1139/a95-009 |
| Bird | *Schiffornis turdina* | 250 | Gap-crossing | Offerman, H.L., Dale, V.H., Pearson, S.M., Bierregaard, R.O., O'Neill, R.V., 1995. Effects of forest fragmentation on neotropical fauna: current research and data availability. Environ. Rev. 3, 191-211. https://doi.org/10.1139/a95-009 |
| Bird | *Tolmomyias assimilis* | 250 | Gap-crossing | Offerman, H.L., Dale, V.H., Pearson, S.M., Bierregaard, R.O., O'Neill, R.V., 1995. Effects of forest fragmentation on neotropical fauna: current research and data availability. Environ. Rev. 3, 191-211. https://doi.org/10.1139/a95-009 |
| Monkey | *Sapajus gr. apella* | 250 | Gap-crossing | Offerman, H.L., Dale, V.H., Pearson, S.M., Bierregaard, R.O., O'Neill, R.V., 1995. Effects of forest fragmentation on neotropical fauna: current research and data availability. Environ. Rev. 3, 191-211. https://doi.org/10.1139/a95-009 |
| Frog | *Rhinella dapsilis* | 300 | Gap-crossing | Offerman, H.L., Dale, V.H., Pearson, S.M., Bierregaard, R.O., O'Neill, R.V., 1995. Effects of forest fragmentation on neotropical fauna: current research and data availability. Environ. Rev. 3, 191-211. https://doi.org/10.1139/a95-009 |
| Frog | *Chiasmocleis shudikarensis* | 300 | Gap-crossing | Offerman, H.L., Dale, V.H., Pearson, S.M., Bierregaard, R.O., O'Neill, R.V., 1995. Effects of forest fragmentation on neotropical fauna: current research and data availability. Environ. Rev. 3, 191-211. https://doi.org/10.1139/a95-009 |
| Frog | *Colostethus marchesianus* | 300 | Gap-crossing | Offerman, H.L., Dale, V.H., Pearson, S.M., Bierregaard, R.O., O'Neill, R.V., 1995. Effects of forest fragmentation on neotropical fauna: current research and data availability. Environ. Rev. 3, 191-211. https://doi.org/10.1139/a95-009 |
| Frog | *Leptodactylus rhodomystax* | 300 | Gap-crossing | Offerman, H.L., Dale, V.H., Pearson, S.M., Bierregaard, R.O., O'Neill, R.V., 1995. Effects of forest fragmentation on neotropical fauna: current research and data availability. Environ. Rev. 3, 191-211. https://doi.org/10.1139/a95-009 |
| Frog | *Osteocephalus buckleyi* | 300 | Gap-crossing | Offerman, H.L., Dale, V.H., Pearson, S.M., Bierregaard, R.O., O'Neill, R.V., 1995. Effects of forest fragmentation on neotropical fauna: current research and data availability. Environ. Rev. 3, 191-211. https://doi.org/10.1139/a95-009 |
| Frog | *Phyllomedusa bicolor* | 300 | Gap-crossing | Offerman, H.L., Dale, V.H., Pearson, S.M., Bierregaard, R.O., O'Neill, R.V., 1995. Effects of forest fragmentation on neotropical fauna: current research and data availability. Environ. Rev. 3, 191-211. https://doi.org/10.1139/a95-009 |
| Bird | *Pithys albifrons* | 300 | Gap-crossing | Offerman, H.L., Dale, V.H., Pearson, S.M., Bierregaard, R.O., O'Neill, R.V., 1995. Effects of forest fragmentation on neotropical fauna: current research and data availability. Environ. Rev. 3, 191-211. https://doi.org/10.1139/a95-009 |
| Bird | *Thamnomanes caesius* | 300 | Gap-crossing | Offerman, H.L., Dale, V.H., Pearson, S.M., Bierregaard, R.O., O'Neill, R.V., 1995. Effects of forest fragmentation on neotropical fauna: current research and data availability. Environ. Rev. 3, 191-211. https://doi.org/10.1139/a95-009 |
| Bird | *Tunchiornis ochraceiceps* | 300 | Gap-crossing | Offerman, H.L., Dale, V.H., Pearson, S.M., Bierregaard, R.O., O'Neill, R.V., 1995. Effects of forest fragmentation on neotropical fauna: current research and data availability. Environ. Rev. 3, 191-211. https://doi.org/10.1139/a95-009 |
| Bird | *Glyphorynchus spirurus* | 400 | Gap-crossing | Offerman, H.L., Dale, V.H., Pearson, S.M., Bierregaard, R.O., O'Neill, R.V., 1995. Effects of forest fragmentation on neotropical fauna: current research and data availability. Environ. Rev. 3, 191-211. https://doi.org/10.1139/a95-009 |
| Bird | *Formicarius colma* | 400 | Gap-crossing | Offerman, H.L., Dale, V.H., Pearson, S.M., Bierregaard, R.O., O'Neill, R.V., 1995. Effects of forest fragmentation on neotropical fauna: current research and data availability. Environ. Rev. 3, 191-211. https://doi.org/10.1139/a95-009 |
| Bird | *Ramphastos tucanus* | 400 | Gap-crossing | Lees, A.C., Peres, C.A., 2009. Gap-crossing movements predict species occupancy in Amazonian forest fragments. Oikos 118, 280-290. https://doi.org/10.1111/j.1600-0706.2008.16842.x |
| Bird | *Ara severa* | 425 | Gap-crossing | Lees, A.C., Peres, C.A., 2009. Gap-crossing movements predict species occupancy in Amazonian forest fragments. Oikos 118, 280-290. https://doi.org/10.1111/j.1600-0706.2008.16842.x |
| Bird | *Pionus menstruus* | 425 | Gap-crossing | Lees, A.C., Peres, C.A., 2009. Gap-crossing movements predict species occupancy in Amazonian forest fragments. Oikos 118, 280-290. https://doi.org/10.1111/j.1600-0706.2008.16842.x |
| Bird | *Amazona ochrocephala* | 425 | Gap-crossing | Lees, A.C., Peres, C.A., 2009. Gap-crossing movements predict species occupancy in Amazonian forest fragments. Oikos 118, 280-290. https://doi.org/10.1111/j.1600-0706.2008.16842.x |
| Bird | *Melanerpes cruentatus* | 425 | Gap-crossing | Lees, A.C., Peres, C.A., 2009. Gap-crossing movements predict species occupancy in Amazonian forest fragments. Oikos 118, 280-290. https://doi.org/10.1111/j.1600-0706.2008.16842.x |
| Bird | *Pteroglossus castanotis* | 425 | Gap-crossing | Lees, A.C., Peres, C.A., 2009. Gap-crossing movements predict species occupancy in Amazonian forest fragments. Oikos 118, 280-290. https://doi.org/10.1111/j.1600-0706.2008.16842.x |
| Bird | *Phaethornis ruber* | 425 | Gap-crossing | Lees, A.C., Peres, C.A., 2009. Gap-crossing movements predict species occupancy in Amazonian forest fragments. Oikos 118, 280-290. https://doi.org/10.1111/j.1600-0706.2008.16842.x |
| Bird | *Dryocopus lineatus* | 425 | Gap-crossing | Lees, A.C., Peres, C.A., 2009. Gap-crossing movements predict species occupancy in Amazonian forest fragments. Oikos 118, 280-290. https://doi.org/10.1111/j.1600-0706.2008.16842.x |
| Bird | *Ara ararauna* | 425 | Gap-crossing | Lees, A.C., Peres, C.A., 2009. Gap-crossing movements predict species occupancy in Amazonian forest fragments. Oikos 118, 280-290. https://doi.org/10.1111/j.1600-0706.2008.16842.x |
| Bird | *Psittacara leucophthalmus* | 425 | Gap-crossing | Lees, A.C., Peres, C.A., 2009. Gap-crossing movements predict species occupancy in Amazonian forest fragments. Oikos 118, 280-290. https://doi.org/10.1111/j.1600-0706.2008.16842.x |
| Bird | *Myrmotherula longipennis* | 700 | Gap-crossing | Offerman, H.L., Dale, V.H., Pearson, S.M., Bierregaard, R.O., O'Neill, R.V., 1995. Effects of forest fragmentation on neotropical fauna: current research and data availability. Environ. Rev. 3, 191-211. https://doi.org/10.1139/a95-009 |
| Bird | *Mionectes macconnelli* | 700 | Gap-crossing | Offerman, H.L., Dale, V.H., Pearson, S.M., Bierregaard, R.O., O'Neill, R.V., 1995. Effects of forest fragmentation on neotropical fauna: current research and data availability. Environ. Rev. 3, 191-211. https://doi.org/10.1139/a95-009 |
| Bird | *Rhynchocyclus olivaceus* | 700 | Gap-crossing | Offerman, H.L., Dale, V.H., Pearson, S.M., Bierregaard, R.O., O'Neill, R.V., 1995. Effects of forest fragmentation on neotropical fauna: current research and data availability. Environ. Rev. 3, 191-211. https://doi.org/10.1139/a95-009 |
| Frog | *Phyllomedusa tarsius* | 1000 | Gap-crossing | Offerman, H.L., Dale, V.H., Pearson, S.M., Bierregaard, R.O., O'Neill, R.V., 1995. Effects of forest fragmentation on neotropical fauna: current research and data availability. Environ. Rev. 3, 191-211. https://doi.org/10.1139/a95-009 |
| Frog | *Phyllomedusa tomopterna* | 1000 | Gap-crossing | Offerman, H.L., Dale, V.H., Pearson, S.M., Bierregaard, R.O., O'Neill, R.V., 1995. Effects of forest fragmentation on neotropical fauna: current research and data availability. Environ. Rev. 3, 191-211. https://doi.org/10.1139/a95-009 |
| Bird | *Gymnopithys rufigula* | 1000 | Gap-crossing | Offerman, H.L., Dale, V.H., Pearson, S.M., Bierregaard, R.O., O'Neill, R.V., 1995. Effects of forest fragmentation on neotropical fauna: current research and data availability. Environ. Rev. 3, 191-211. https://doi.org/10.1139/a95-009 |
| Bird | *Percnostola rufifrons* | 1000 | Gap-crossing | Offerman, H.L., Dale, V.H., Pearson, S.M., Bierregaard, R.O., O'Neill, R.V., 1995. Effects of forest fragmentation on neotropical fauna: current research and data availability. Environ. Rev. 3, 191-211. https://doi.org/10.1139/a95-009 |
| Bird | *Turdus albicollis* | 1000 | Gap-crossing | Offerman, H.L., Dale, V.H., Pearson, S.M., Bierregaard, R.O., O'Neill, R.V., 1995. Effects of forest fragmentation on neotropical fauna: current research and data availability. Environ. Rev. 3, 191-211. https://doi.org/10.1139/a95-009 |
| Plant | *Astrocaryum aculeatum* | 194 | Pollen dispersal | Ramos, S.L.F., Dequigiovanni, G., Sebbenn, A.M., Lopes, M.T.G., Kageyama, P.Y., Macêdo, J.L.V., Kirst, M., Veasey, E.A., 2016. Spatial genetic structure, genetic diversity and pollen dispersal in a harvested population of Astrocaryum aculeatum in the Brazilian Amazon. BMC Genet 17, 63. https://doi.org/10.1186/s12863-016-0371-8 |
| Plant | *Bertholletia excelsa* | 372 | Pollen dispersal | Martins, K., Santos, R.S.O., Campos, T., Wadt, L.H.O., 2018. Pollen and seed dispersal of Brazil nut trees in the southwestern Brazilian Amazon. Acta Amaz. 48, 217-223. https://doi.org/10.1590/1809-4392201800021 |
| Plant | *Carapa guianensis* | 430 | Pollen dispersal | Martins, K., Raposo, A., Klimas, C.A., Veasey, E.A., Kainer, K., Wadt, L.H.O., 2012. Pollen and seed flow patterns of *Carapa guianensis* Aublet. (Meliaceae) in two types of Amazonian forest. Genet. Mol. Biol. 35, 818-826. https://doi.org/10.1590/S1415-47572012005000068 |
| Plant | *Swietenia macrophylla* | 576 | Pollen dispersal | Sebbenn, A.M., Licona, J.C., Mostacedo, B., Degen, B., 2012. Gene flow in an overexploited population of *Swietenia macrophylla* King (Meliaceae) in the Bolivian Amazon. Silvae Genet. 61, 212-220. https://doi.org/10.1515/sg-2012-0027 |
| Plant | *Bagassa guianensis* | 961 | Pollen dispersal | Silva, M.B., Kanashiro, M., Ciampi, A.Y., Thompson, I., Sebbenn, A.M., 2008. Genetic effects of selective logging and pollen gene flow in a low-density population of the dioecious tropical tree *Bagassa guianensis* in the Brazilian Amazon. For. Ecol. Manag. 255: 1548-1558. https://doi.org/10.1016/j.foreco.2007.11.012 |
| Plant | *Euterpe precatoria* | 1544 | Pollen dispersal | Ramos, S.L.F., Dequigiovanni, G., Sebbenn, A.M., Lopes, M.T.G., Macedo, J.L.V., Veasey, E.A., Alves-Pereira, A., Silva, P.P., Garcia, J.N., Kageyama, P.Y., 2018. Paternity analysis, pollen flow, and spatial genetic structure of a natural population of *Euterpe precatoria* in the Brazilian Amazon. Ecol. Evol. 8, 11143-11157. https://doi.org/10.1002/ece3.4582 |
| Plant | *Hymenaea courbaril* | 2204 | Pollen dispersal | Carneiro, F.S., 2010. Efeito do corte seletivo de árvores sobre o sistema de reprodução e dispersão de pólen em população de *Hymenaea courbaril* na Amazônia Brasileira. Dissertação. Universidade Estadual Paulista Julio de Mesquita Filho. 76p. |
| Plant | *Bagassa guianensis* | 2637 | Pollen dispersal | Arruda, C.C.B., Silva, M.B., Gribel, R., Lemes, M.R., Kanashiro, M., Sebbenn, A.M., 2015. Logging decreases the pollen dispersal distance in a low-density population of the tree *Bagassa guianensis* in the Brazilian Amazon. Silvae Genet. 64, 279-290. https://doi.org/10.1515/sg-2015-0026 |
| Plant | *Symphonia globulifera* | 2832 | Pollen dispersal | Carneiro, F.C., Degen, B., Kanashiro, M., Lacerda, A.E.B., Sebbenn, A.M., 2009. High levels of pollen dispersal detected through paternity analysis from a continuous *Symphonia globulifera* population in the Brazilian Amazon. For. Ecol. Manag. 258, 1260-1266. https://doi.org/10.1016/j.foreco.2009.06.019 |
| Plant | *Marcgravia longifolia* | 1350 | Pollen dispersal | Gottstein, M.; Thiel, S.; Vornhagen, J.L.; Mengel, C.; Tschapka, M.; Heymann, E.W.; Heer, K. 2025. Gene flow and vertical stratification of pollination in the bat-pollinated liana *Marcgravia longifolia*. Ecology and Evolution 15: e72050. https://doi.org/10.1002/ece3.72050 |
| Plant | *Dinizia excelsa* | 3200 | Pollen dispersal | Dick, C.W. 2001. Genetic rescue of remnant tropical trees by an alien pollinator. Proc. R. Soc. Lond. Ser. B. 268: 2391-2396. https://doi.org/10.1098/rspb.2001.1781 |
| Bee | *Apis mellifera* | 3200 | Pollen dispersal | Dick, C.W. 2001. Genetic rescue of remnant tropical trees by an alien pollinator. Proc. R. Soc. Lond. Ser. B. 268: 2391-2396. https://doi.org/10.1098/rspb.2001.1781 |
| Plant | *Guadua weberbaueri* | 156 | Pollen/seed dispersal | Leal, G.S.A., Leal, F.A., Gomes, H.T., Souza, A.M., Ribeiro, S.C., Scherwinski-Pereira, J.E., 2021. Structure and genetic diversity of natural populations of *Guadua weberbaueri* in the southwestern Amazon, Brazil. J. For. Res. 32, 755-763. https://doi.org/10.1007/s11676-020-01128-4 |
| Plant | *Ormosia costulada* | 0 | Seed dispersal | Macedo, M., 1977. Dispersão de plantas lenhosas de uma campina amazônica. Acta Amazônica 7, 69p. https://doi.org/10.1590/1809-43921977071s005 |
| Plant | *Vernonia grisea* | 7 | Seed dispersal | Macedo, M., 1977. Dispersão de plantas lenhosas de uma campina amazônica. Acta Amazônica 7, 69p. https://doi.org/10.1590/1809-43921977071s005 |
| Plant | *Borreria capitata var. tenella* | 11 | Seed dispersal | Macedo, M., 1977. Dispersão de plantas lenhosas de uma campina amazônica. Acta Amazônica 7, 69p. https://doi.org/10.1590/1809-43921977071s005 |
| Plant | *Bocageopsis multiflora* | 31 | Seed dispersal | Cramer, J.M., Mesquita, R.C.G., Williamson, G.B., 2007. Forest fragmentation differentially affects seed dispersal of large and small-seeded tropical trees. Biol. Conserv. 137, 415-423. https://doi.org/10.1016/j.biocon.2007.02.019 |
| Plant | *Duckeodendron cestroides* | 40 | Seed dispersal | Cramer, J.M., Mesquita, R.C.G., Williamson, G.B., 2007. Forest fragmentation differentially affects seed dispersal of large and small-seeded tropical trees. Biol. Conserv. 137, 415-423. https://doi.org/10.1016/j.biocon.2007.02.019 |
| Plant | *Bertholletia excelsa* | 50 | Seed dispersal | Haugassen, J.M.T., Haugaasen, T., Peres, C.A., Gribel, R., Wegge, P., 2010. Seed dispersal of the Brazil nut tree (*Bertholletia excelsa*) by scatter-hoarding rodents in a central Amazonian forest. J. Trop. Ecol. 26, 251-262. DOI:10.1017/S0266467410000027 |
| Plant | *Theobroma speciosum* | 70 | Seed dispersal | Dardengo, J.F.G., Rossi, A.A.B., Silva, B.M., Silva, I.V., Silva, C.J., Sebbenn, A.M., 2016. Diversity and spatial genetic structure of a natural population of *Theobroma speciosum* (Malvaceae) in the Brazilian Amazon. Rev. Biol. Trop. 64, 1091-1099. http://dx.doi.org/10.15517/rbt.v64i3.21461. |
| Plant | *Pouteria cf. bilocularis* | 75 | Seed dispersal | Julliot, C., 1996. Seed dispersal by Red Howling Monkey s (*Alouatta seniculus*) in the Tropical Rain Forest of French Guiana. Int. J. Primatol. 17, 239-258. https://doi.org/10.1007/BF02735451 |
| Plant | *Ecclinusa lanceolata* | 97 | Seed dispersal | Julliot, C., 1996. Seed dispersal by Red Howling Monkey s (*Alouatta seniculus*) in the Tropical Rain Forest of French Guiana. Int. J. Primatol. 17, 239-258. https://doi.org/10.1007/BF02735451 |
| Plant | *Tapirira peckoltiana* | 115 | Seed dispersal | Julliot, C., 1996. Seed dispersal by Red Howling Monkey s (*Alouatta seniculus*) in the Tropical Rain Forest of French Guiana. Int. J. Primatol. 17, 239-258. https://doi.org/10.1007/BF02735451 |
| Plant | *Swietenia macrophylla* | 155 | Seed dispersal | Norghauer, J.M., Nock, C.A., Grogan, J., 2011. The importance of tree size and fecundity for wind dispersal of Big-leaf Mahogany. PLoS One 6, e17488. https://doi.org/10.1371/journal.pone.0017488 |
| Plant | *Pouteria aff. hispida* | 180 | Seed dispersal | Julliot, C., 1996. Seed dispersal by Red Howling Monkey s (*Alouatta seniculus*) in the Tropical Rain Forest of French Guiana. Int. J. Primatol. 17, 239-258. https://doi.org/10.1007/BF02735451 |
| Plant | *Salacia cf. cordata* | 200 | Seed dispersal | Julliot, C., 1996. Seed dispersal by Red Howling Monkey s (*Alouatta seniculus*) in the Tropical Rain Forest of French Guiana. Int. J. Primatol. 17, 239-258. https://doi.org/10.1007/BF02735451 |
| Bird | *Schiffornis turdina* | 200 | Seed dispersal | Santana, F.D., 2017. Dispersão de sementes de Marantaceae: o papel da heterogeneidade ambiental na interações planta-dispersor e nos padrões de distribuição de ervas de sub-bosque na Amazônia Central. Tese. Instituto Nacional de Pesquisas da Amazônia. 112p. |
| Plant | *Bagassa guianensis* | 231 | Seed dispersal | Julliot, C., 1996. Seed dispersal by Red Howling Monkey s (*Alouatta seniculus*) in the Tropical Rain Forest of French Guiana. Int. J. Primatol. 17, 239-258. https://doi.org/10.1007/BF02735451 |
| Plant | *Chrysophyllum prieurri* | 242 | Seed dispersal | Julliot, C., 1996. Seed dispersal by Red Howling Monkey s (*Alouatta seniculus*) in the Tropical Rain Forest of French Guiana. Int. J. Primatol. 17, 239-258. https://doi.org/10.1007/BF02735451 |
| Monkey | *Ateles paniscus* | 254 | Seed dispersal | Zhang, S.Y., Wang, L.X., 1995. Fruit consumption and seed dispersal of *Ziziphus cinnamomum* (Rhamnaceae) by two sympatric primates (*Cebus apella* and *Ateles paniscus*) in French Guiana. Biotropica 27, 397-401. https://doi.org/10.2307/2388926 |
| Plant | *Cecropia obtusa* | 255 | Seed dispersal | Julliot, C., 1996. Seed dispersal by Red Howling Monkey s (*Alouatta seniculus*) in the Tropical Rain Forest of French Guiana. Int. J. Primatol. 17, 239-258. https://doi.org/10.1007/BF02735451 |
| Plant | *Doliocarpus sp.* | 268 | Seed dispersal | Julliot, C., 1996. Seed dispersal by Red Howling Monkey s (*Alouatta seniculus*) in the Tropical Rain Forest of French Guiana. Int. J. Primatol. 17, 239-258. https://doi.org/10.1007/BF02735451 |
| Plant | *Pouteria* sp*.* | 270 | Seed dispersal | Julliot, C., 1996. Seed dispersal by Red Howling Monkey s (*Alouatta seniculus*) in the Tropical Rain Forest of French Guiana. Int. J. Primatol. 17, 239-258. https://doi.org/10.1007/BF02735451 |
| Tortoise | *Chelonoidis denticulata* | 300 | Seed dispersal | Jerozolimski, A., Ribeiro, M.B.N., Martins, M., 2009. Are tortoises important seed dispersers in Amazonian forests? Oecologia 161, 517-528. https://doi.org/10.1007/s00442-009-1396-8 |
| Plant | *Pouteria ambelaniifolia* | 318 | Seed dispersal | Julliot, C., 1996. Seed dispersal by Red Howling Monkey s (*Alouatta seniculus*) in the Tropical Rain Forest of French Guiana. Int. J. Primatol. 17, 239-258. https://doi.org/10.1007/BF02735451 |
| Plant | *Carapa guianensis* | 334 | Seed dispersal | Martins, K., Raposo, A., Klimas, C.A., Veasey, E.A., Kainer, K., Wadt, L.H.O., 2012. Pollen and seed flow patterns of *Carapa guianensis* Aublet. (Meliaceae) in two types of Amazonian forest. Genet. Mol. Biol. 35, 818-826. https://doi.org/10.1590/S1415-47572012005000068 |
| Plant | *Micropholis obscura* | 343 | Seed dispersal | Julliot, C., 1996. Seed dispersal by Red Howling Monkey s (*Alouatta seniculus*) in the Tropical Rain Forest of French Guiana. Int. J. Primatol. 17, 239-258. https://doi.org/10.1007/BF02735451 |
| Plant | *Pouteria egregia* | 343 | Seed dispersal | Julliot, C., 1996. Seed dispersal by Red Howling Monkey s (*Alouatta seniculus*) in the Tropical Rain Forest of French Guiana. Int. J. Primatol. 17, 239-258. https://doi.org/10.1007/BF02735451 |
| Plant | *Talisia* sp*.* | 347 | Seed dispersal | Julliot, C., 1996. Seed dispersal by Red Howling Monkey s (*Alouatta seniculus*) in the Tropical Rain Forest of French Guiana. Int. J. Primatol. 17, 239-258. https://doi.org/10.1007/BF02735451 |
| Monkey | *Sapajus* gr*. apella* | 390 | Seed dispersal | Zhang, S.Y., Wang, L.X., 1995. Fruit consumption and seed dispersal of *Ziziphus cinnamomum* (Rhamnaceae) by two sympatric primates (*Cebus apella* and *Ateles paniscus*) in French Guiana. Biotropica 27, 397-401. https://doi.org/10.2307/2388926 |
| Plant | *Carapa guianensis* | 397 | Seed dispersal | Martins, K., Raposo, A., Klimas, C.A., Veasey, E.A., Kainer, K., Wadt, L.H.O., 2012. Pollen and seed flow patterns of *Carapa guianensis* Aublet. (Meliaceae) in two types of Amazonian forest. Genet. Mol. Biol. 35, 818-826. https://doi.org/10.1590/S1415-47572012005000068 |
| Plant | *Dendrobangia boliviana* | 459 | Seed dispersal | Julliot, C., 1996. Seed dispersal by Red Howling Monkey s (*Alouatta seniculus*) in the Tropical Rain Forest of French Guiana. Int. J. Primatol. 17, 239-258. https://doi.org/10.1007/BF02735451 |
| Plant | *Swartzia dolicopoda* | 500 | Seed dispersal | Macedo, M., 1977. Dispersão de plantas lenhosas de uma campina amazônica. Acta Amazônica 7, 69p. https://doi.org/10.1590/1809-43921977071s005 |
| Bird | *Hylexetastes perrotti* | 520 | Seed dispersal | Santana, F.D., 2017. Dispersão de sementes de Marantaceae: o papel da heterogeneidade ambiental na interações planta-dispersor e nos padrões de distribuição de ervas de sub-bosque na Amazônia Central. Tese. Instituto Nacional de Pesquisas da Amazônia. 112p. |
| Bird | *Momotus momota* | 540 | Seed dispersal | Santana, F.D., 2017. Dispersão de sementes de Marantaceae: o papel da heterogeneidade ambiental na interações planta-dispersor e nos padrões de distribuição de ervas de sub-bosque na Amazônia Central. Tese. Instituto Nacional de Pesquisas da Amazônia. 112p. |
| Plant | *Parahancornia fasciculata* | 555 | Seed dispersal | Julliot, C., 1996. Seed dispersal by Red Howling Monkey s (*Alouatta seniculus*) in the Tropical Rain Forest of French Guiana. Int. J. Primatol. 17, 239-258. https://doi.org/10.1007/BF02735451 |
| Monkey | *Alouatta seniculus* | 555 | Seed dispersal | Julliot, C., 1996. Seed dispersal by Red Howling Monkey s (*Alouatta seniculus*) in the Tropical Rain Forest of French Guiana. Int. J. Primatol. 17, 239-258. https://doi.org/10.1007/BF02735451 |
| Monkey | *Saguinus* spp*.* | 599 | Seed dispersal | Garber, P.A.,1986. The ecology of seed dispersal in two species of Callitrichid Primates (*Sanguinus mystax* and *Saguinus fuscicollis*). Am. J. Primatol. 10, 155-170. DOI: 10.1002/ajp.1350100206 |
| Monkey | *Alouatta seniculus* | 637 | Seed dispersal | Yumoto, T., Kimura, K., Nishimura, A., 1999. Estimation of the retention times and distances of seed dispersed by two monkey species, *Alouatta seniculus* and *Lagothrix lagotricha*, in Colombian forest. Ecol. Res. 14, 179-191. https://doi.org/10.1046/j.1440-1703.1999.00286.x |
| Plant | *Bertholletia excelsa* | 655 | Seed dispersal | Martins, K., Santos, R.S.O., Campos, T., Wadt, L.H.O., 2018. Pollen and seed dispersal of Brazil nut trees in the southwestern Brazilian Amazon. Acta Amaz. 48, 217-223. https://doi.org/10.1590/1809-4392201800021 |
| Monkey | *Lagothrix lagotricha* | 1106 | Seed dispersal | Yumoto, T., Kimura, K., Nishimura, A. 1999. Estimation of the retention times and distances of seed dispersed by two monkey species, *Alouatta seniculus* and *Lagothrix lagotricha*, in Colombian forest. Ecol. Res. 14, 179-191. https://doi.org/10.1046/j.1440-1703.1999.00286.x |
| Monkey | *Saguinus bicolor* | 1170 | Seed dispersal | Lima, R.G.F., 2012. Dispersão de sementes por sauins-de-coleira (*Saguinus bicolor* Spix, 1823, Primates: Cebidae): efeitos do padrão de deslocamento na formação de sombras de sementes. Dissertação. Universidade Federal do Pará. 50p. |
| Bird | *Ara glaucogularis* | 1200 | Seed dispersal | Baños-Villalba, A., Blanco, G., Díaz-Luque, J.A., Dénes, F.V., Hiraldo, F., Tella, J.L., 2017. Seed dispersal by macaws shapes the landscape of an Amazonian ecosystem. Sci. Rep. 7, 7373. https://doi.org/10.1038/s41598-017-07697-5 |
| Bird | *Psophia crepitans* | 1897 | Seed dispersal | Santana, F.D., 2017. Dispersão de sementes de Marantaceae: o papel da heterogeneidade ambiental na interações planta-dispersor e nos padrões de distribuição de ervas de sub-bosque na Amazônia Central. Tese. Instituto Nacional de Pesquisas da Amazônia. 112p. |
| Plant | *Maximiliana maripa* | 2000 | Seed dispersal | Fragoso, J.M.V., 1997. Tapir-generated seed shadows: scale-dependent patchiness in the Amazon rain forest. J. Ecol. 85, 519-529. |
| Lowland tapir | *Tapirus terrestris* | 2000 | Seed dispersal | Fragoso, J.M.V., 1997. Tapir-generated seed shadows: scale-dependent patchiness in the Amazon rain forest. J. Ecol. 85, 519-529. |
| Bird | *Tinamus major* | 2018 | Seed dispersal | Santana, F.D., 2017. Dispersão de sementes de Marantaceae: o papel da heterogeneidade ambiental na interações planta-dispersor e nos padrões de distribuição de ervas de sub-bosque na Amazônia Central. Tese. Instituto Nacional de Pesquisas da Amazônia. 112p. |
